# Innate Immune Training of Human Macrophages by Cathelicidin Analogs

**DOI:** 10.3389/fimmu.2022.777530

**Published:** 2022-07-26

**Authors:** Albert van Dijk, Jennifer Anten, Anne Bakker, Noah Evers, Anna T. Hoekstra, Jung-Chin Chang, Maaike R. Scheenstra, Edwin J. A. Veldhuizen, Mihai G. Netea, Celia R. Berkers, Henk P. Haagsman

**Affiliations:** ^1^ Division Infectious Diseases and Immunology, Department Biomolecular Health Sciences, Faculty of Veterinary Medicine, Utrecht University, Utrecht, Netherlands; ^2^ Biomolecular Mass Spectrometry and Proteomics, Bijvoet Center for Biomolecular Research, Utrecht University, Utrecht, Netherlands; ^3^ Division Cell Biology, Metabolism & Cancer, Department Biomolecular Health Sciences, Faculty of Veterinary Medicine, Utrecht University, Utrecht, Netherlands; ^4^ Department of Internal Medicine, Radboud Center for Infectious Diseases (RCI), Radboud University Nijmegen Medical Centre, Nijmegen, Netherlands

**Keywords:** host defense peptide, cathelicidin, macrophage, trained immunity, immunomodulation, metabolomics, Seahorse analysis

## Abstract

Trained innate immunity can be induced in human macrophages by microbial ligands, but it is unknown if exposure to endogenous alarmins such as cathelicidins can have similar effects. Previously, we demonstrated sustained protection against infection by the chicken cathelicidin-2 analog DCATH-2. Thus, we assessed the capacity of cathelicidins to induce trained immunity. PMA-differentiated THP-1 (dTHP1) cells were trained with cathelicidin analogs for 24 hours and restimulated after a 3-day rest period. DCATH-2 training of dTHP-1 cells amplified their proinflammatory cytokine response when restimulated with TLR2/4 agonists. Trained cells displayed a biased cellular metabolism towards mTOR-dependent aerobic glycolysis and long-chain fatty acid accumulation and augmented microbicidal activity. DCATH-2-induced trained immunity was inhibited by histone acetylase inhibitors, suggesting epigenetic regulation, and depended on caveolae/lipid raft-mediated uptake, MAPK p38 and purinergic signaling. To our knowledge, this is the first report of trained immunity by host defense peptides.

## Introduction

Innate immune memory or “trained immunity” has been shown to be induced by various microbial components in NK cells and monocytes/macrophages (1–4). Bacterial, fungal and viral ligands can reprogram the monocyte phenotype *via* activation of pattern recognition receptors (PRR) towards an enhanced (trained) or diminished (tolerance) immune response to restimulation. Previous exposure to heat-killed *Candida albicans* or the fungal cell wall component β-glucan was shown to generate protection against re-infection in mice in a macrophage dependent manner, through an amplification of the pro-inflammatory cytokine response to TLR2 and TLR4 ligands (2). In addition to β-glucans, *in vitro* trained immunity can be elicited in monocytes/macrophages by components of Gram-positive bacteria (muramyl dipeptide) (2, 3), BCG (Bacille Calmette-Guérin) (3), low doses of polysaccharides (1) and oxidized low-density lipoproteins (5). The mechanisms underlying trained immunity are a rewired cell metabolism towards aerobic glycolysis and changes in the epigenetic landscape at specific loci containing immune-related genes (6). However, these effects are dependent on the length of the priming time (2) and are dependent on ligand concentration. High amounts of fungal cell wall β-glucans (1 µg/ml), peptidoglycan components Tri-DAP (10 µg/ml) and muramyl dipeptide (10 µg/ml), acting *via* dectin-1, NOD1 and NOD2 receptors, respectively, were found to induce a trained immune response in monocytes. Most TLR ligands induce tolerance at high doses, while at low concentrations tolerance is absent, and in some cases, e.g., LPS and flagellin, trained immunity is induced (1). We hypothesized that host-derived molecules or compounds mimicking these molecules could function as danger signals that prepare host cells for an amplified response to microbial exposure.

Cathelicidins are host defence peptides (HDPs), part of the innate immune system (7) and can be considered as “alarmins”, endogenous proteins and peptides that are passively (necrosis) or actively released through microbial exposure or neutrophil and mast cell degranulation upon tissue injury or infection (8). Potent immunomodulatory effects on macrophages have been reported for human cathelicidin LL-37 and chicken CATH-2 *in vitro* (9–12). *In vivo*, antimicrobial efficacy of cathelicidin-derived peptides was demonstrated in mouse infection models for invasive *Staphylococcus aureus* (13, 14), MRSA (15), *Escherichia coli* (13), and *Mycobacterium tuberculosis* (14) infection. To increase the therapeutic potential of cathelicidin-derived peptides, we used a full D-amino acid analog to gain high resistance against proteases while maintaining low immunogenicity (16). We found that prophylactic treatment of chicken embryos by *in ovo* injection with a low dose (1 mg/kg) DCATH-2 considerably reduced colibacillosis-associated mortality and morbidity (17). Similarly, delayed mortality was also observed when very low doses (2.6 ng/kg) of DCATH-2 were injected into the yolk of zebrafish embryos followed by intravenously infection with a lethal dose of *Salmonella enterica* (18). These findings suggest a generic mechanism of action of CATH-2 analogs across species. Elucidation of the mechanism of action may aid the development of alternatives to conventional antibiotics that boost resistance against infectious diseases in multiple species.

Interestingly, the peptide doses used in the latter studies were far too low to explain the protective effects by a direct antimicrobial action of the peptide and underline that the *in vivo* protective effects of DCATH-2 are of an immunomodulatory nature. Furthermore, the 10-day gap between *in ovo* injection of DCATH-2 in chicken embryos and inoculation with *E. coli* implies that a heterologous memory effect must play a role. Importantly, *in ovo* DCATH-2 treatment of chicken embryos in the absence of infection did not result in major changes in the functionality and numbers of peripheral blood cell populations (19). The lack of visible peptide-induced changes in immune cell functionality in the absence of infection, and improved resistance of peptide-treated animals *in vivo* when challenged with a bacterial infection led us to hypothesize that these events may be explained by peptide-induced trained immunity. The objective of this study was to examine the capacity of cathelicidins to train macrophages. We demonstrate here that cathelicidin analogs induced trained immunity in macrophages leading to enhanced proinflammatory cytokine production upon repeated stimulation. Understanding the exact mechanisms underlying cathelicidin-mediated trained immunity is an important step in the comprehension of the alarmin functions of cathelicidins during infection and inflammation, and for further development of cathelicidin-based therapeutics as an alternative to conventional antibiotics.

## Material and Methods

### Reagents and Cell Line

*Salmonella enterica subsp. enterica* serovar minnesota R595 LPS (InvivoGen, Toulouse, France), *E. coli* serotype O111:B4 LPS (InvivoGen), Pam3CSK4 (InvivoGen), Pam2CSK4 (InvivoGen), phorbol 12-myristate 13-acetate (Sigma-Aldrich, Saint Louis, MO, USA), mTOR inhibitor rapamycin (Sigma-Aldrich), PI3K inhibitor wortmannin (InvivoGen), Akt inhibitors triciribine and AZD5363 (SelleckChem, Houston, TX, USA), HIF-1α inhibitor ascorbate (Sigma-Aldrich), AMPK activators 5-aminoimidazole-4-carboxamide ribonucleotide (AICAR; Sigma-Aldrich), and metformin (InvivoGen), MAPK p38 inhibitor SB203580 (InvivoGen), MAPK ERK inhibitor PD98059 (Sigma-Aldrich), JNK inhibitor SP600125 (Sigma-Aldrich), histone acetyl transferase inhibitors epigallocatechin gallate (EGCG, Sigma-Aldrich), anacardic acid (Sigma-Aldrich), curcumin (Sigma-Aldrich), and garcinol (Enzo Life Sciences, NY, USA), NFκB inhibitor Bay-11-7085 (InvivoGen), histone methyltransferase inhibitor (MTA, Sigma-Aldrich), HMTase inhibitor MM-102 (SelleckChem), P2X/P2Y receptor inhibitor suramin (Sigma-Aldrich), P2X7 receptor inhibitor KN-62 (Sigma-Aldrich), lipid raft/caveola-mediated endocytosis inhibitor nystatin (Sigma-Aldrich), clathrin-mediated endocytosis inhibitor chlorpromazine (Sigma-Aldrich). THP-1 (human monocytic leukemia cell line) cells were purchased from the American Type Culture Center (ATCC, Manassas, VA, USA). Oligomycin A, FCCP, Antimycin A, Rotenone, 2-deoxyglucose, and DMEM powder (without glucose, L-glutamine, phenol red, sodium pyruvate and sodium bicarbonate) were all purchased from Sigma-Aldrich. Sodium pyruvate (100 mM solution), MEM non-essential amino acid mixture, glutamax, and penicillin-streptomycin solution were purchased from Gibco.

### Peptides

Peptide CATH-2 (Chicken cathelicidin-2; RFGRFLRKIRRFRPKVTITIQGSARF-NH2), its full D-amino acid analog, DCATH-2 (rfgrflrkirrfrpkvtitiqgsarf-NH2), LL-37 (LLGDFFRKSKEKIGKEFKRIVQRIKDFLRNLVPRTES) and N-terminal TAMRA-labeled DCATH-2 (TD, TAMRA-rfgrflrkirrfrpkvtitiqgsarf-NH2) were synthesized by FMOC chemistry at CPC scientific (San Jose, CA, USA). Peptides were purified to ≥95% by reversed phase HPLC and checked by mass spectrometry. Peptides were dissolved in LPS-free water (WFI; Life technologies, Carlsbad, CA, USA), fluorescently labeled peptide was first dissolved in DMSO before further dilution in cell culture media.

### Stimulation Experiments

THP-1 cells were cultured in Iscove Modified Dulbecco Media (IMDM; Life technologies) containing Glutamax-I, sodium pyruvate, 10% fetal bovine serum at 37°C and 5% CO_2_. For differentiation to macrophage-like cells, THP-1 cells were grown in IMDM/FBS medium containing 8 or 100 nM PMA and seeded in 96 wells (5 × 10^4^ cells/well), 24 wells (3 × 10^5^ cells/well) or 6 wells plates (1 × 10^6^ cells/well) during 48 h. Subsequently, dTHP-1 were washed with pre-warmed IMDM/FBS, left overnight to rest and primed during 1 to 24 h with 2-10 µM peptides of fresh IMDM/FBS medium, washed 3 times in medium. After 3 days rest cells were stimulated with various stimuli: *S. minnesota* LPS (10 ng/ml), *E. coli* LPS (10 or 50 ng/ml), Pam3CSK4 (1µg/ml) or Pam2CSK4 (1 µg/ml). After 24 h supernatants were collected and stored at -20°C. For inhibition, dTHP-1 cells were pre-incubated for 1 h before priming and during priming with rapamycin (10 nM), wortmannin (1 µM), ascorbate (5 µM), AICAR (50 nM), metformin (0.3 mM), triciribine (1 and 5 µM), AZD5363 (1 and 5 µM), SB203580 (10 µM), PD98059 (10 µM), SP600125 (10 µM), EGCG (40 µM), anacardic acid (50 µM), curcumin (10 µM), garcinol (10 µM), MTA (1 mM), MM-102 (25 µM), Bay-11-7085 (10 µM), suramin (50 µM), KN-62 (3 µM), nystatin (10 µg/ml), and chlorpromazine (10 µM).

### ELISAs

TNFα, IL-6, CXCL10 and CCL5 production was measured using ELISA (R&D systems, Minneapolis, MN, USA) following instructions of the manufacturer.

### Antibacterial Activity

Antibacterial activity was determined according to Tang et al. (20). dTHP-1 cells were seeded in 6 well plates and primed with DCATH-2 or medium as described. Log-phase culture of *Salmonella enterica subsp. enterica* serovar enteritidis 706 (Se706) was added to each well at a MOI of 1. After 2 h incubation at 37°C, cells were washed twice with warm Dulbecco’s Phosphate buffered saline (DPBS; Life technologies) and further incubated for 1 h at 37°C with IMDM/FBS medium containing 300 µg/ml colistin (MP biochemicals, Santa Ana, CA, USA). After incubation, cells were washed 3 times in DPBS and the lysed with 1% triton X-100. Well contents were serially diluted in tryptone soy broth (Oxoid, Basingstoke, UK), plated on tryptone soy agar and counted after 24 h at 37°C.

### Candidacidal Activity

dTHP-1 cells were seeded in 6 well plates and primed with DCATH-2 or medium as described. *Candida albicans* ATCC10231 was grown in yeast malt broth (Oxoid) at 30°C, diluted in DPBS to 1 × 10^4^ CFU/ml and added to each well (MOI of 0.03). After 5 h incubation at 30°C, supernatants were transferred and kept. The remaining cells were supplemented with 0.5 ml of sterile water, mixed vigorously and combined with their corresponding well supernatants. Serial dilutions prepared in yeast malt broth were plated onto yeast malt agar. Colonies were counted after 48 h at 30°C.

### Confocal Imaging

3 × 10^5^ dTHP-1 cells were seeded on 8 mm coverslips in 24 wells plates and differentiated for 48 h with 100 nM PMA in 0.5 ml medium. Wells were washed 3 times, and cells were primed for 3 h with TAMRA-labelled DCATH-2 (TD) or medium. For inhibition conditions, cells were pre-incubated before priming for 1 h with 3 µM KN-62, 50 µM suramin, 10 µM chlorpromazine, or 10 µg/ml nystatin. After priming, coverslips were washed 3 times with warm medium followed by fixation for 30 min in 4% paraformaldehyde solution (0.1 M phosphate buffer, pH 7.4). After staining of nuclei with Hoechst (Molecular probes, Eugene, OR, USA) for 10 min, coverslips were mounted on glass slides using ProLong glass antifade mountant (Thermofisher scientific, Waltham, MA, USA). Confocal images were acquired on a Leica SPE-II using the 100x HCX PLAN APO (NA = 1.4-0.7) objective. Imaging was performed using a quadruple band beam splitter for the 561 nm laser. Visualization of Hoechst stained dTHP-1 cells was done with a 405 nm (100 mW) Coherent Violet Cube laser. Image analysis was done using composite images of both channels. In brief, regions of interest were set manually using DIC for segmentation. To isolate puncta from background noise, a threshold was set for the TAMRA channel and intensity and area of puncta were measured partially automated.

### Metabolomics Experiments

dTHP-1 cells were seeded (1 × 10^6^ cells/well) in 6 wells plates and primed during 24 h with 5 µM DCATH-2 as described using medium as control. Cell supernatants, cell lysates and medium controls were collected before and after 24 h priming in the absence and presence of 10 nM rapamycin, after washing followed by 3 days rest and after subsequent 24 h LPS (*E. coli* O111:B4) stimulation. For sample collections, cells were washed once in ice-cold DPBS, lysed with by adding 1 ml cold methanol/acetonitrile/water (2:2:1) lysis buffer, scraped and transferred in to vials. Cell supernatants and medium control samples (10 µl) were directly mixed with 200 µl lysis buffer. Samples were shaken for 10 min at 4°C and centrifuged for 15 min at 18.000 ×g and 4°C. Supernatants were flash frozen in liquid nitrogen and stored at -80°C for analysis. Liquid chromatography-mass spectrometry analysis was performed using an Exactive mass spectrometer (Thermofisher scientific), coupled to a Dionex Ultimate 3000 auto sampler and pump (Thermofisher scientific). The mass spectrometry operated in polarity-switching mode with spray voltages of 4.5 and −3.5 kV. Metabolites were separated using a Sequant ZIC-pHILIC column (2.1 × 150 mm, 5 mm, guard column 2.1 × 20 mm, 5 mm; Merck) using a linear gradient of acetonitrile and eluent A 20 mM (NH_4_)_2_CO_3_, 0.1% NH_4_OH in ULC/MS grade water (Biosolve BV, Valkenswaard, The Netherlands) and a flow rate of 150 µl/min. Metabolites were identified and peak intensities quantified using LCquan software (Thermofisher scientific) on the basis of exact mass within 5 ppm and further validated by concordance with retention times of commercially available standards. Peak intensities were normalized on cell counts of parallel wells before and after LPS stimulation.

### Extracellular Flux Analysis

On day 1, THP-1 cells were seeded at the density of 1.1 × 10^5^ cells per well and differentiated with 100 nM PMA in a XFe24 cell culture plate. On day 3, cells were washed 3 times with culture medium and allowed to rest for 1 day. On day 4, cells were pre-treated with 10 nM rapamycin or control medium, followed by a 24 h treatment of 5 µM DCATH-2 or control medium in the presence and absence of rapamycin. On day 5, cells were washed 3 times with culture medium to completely remove rapamycin and DCATH-2 and incubated in fresh culture medium for 3 days. On day 8, the cells were used for the extracellular flux analysis.

The extracellular acidification rate (ECAR) and the oxygen consumption rate (OCR) were measured using the Seahorse XFe24 Analyzer (Agilent, United States). For the extracellular flux analysis, the culture medium was replaced by the experimental medium: bicarbonate-free DMEM supplemented with 20 mM glucose, 1 mM glutamax, 1 mM sodium pyruvate, 1× non-essential amino acid mixture, 2 mM HEPES-NaOH, 10 U/L penicillin and 10 µg/mL streptomycin, with pH titrated to 7.35-7.40 at room temperature. The culture medium was removed and washed two times with 400 µL experimental medium and further incubated in 250 µL experiment medium for about 1 hour. Before the start of the extracellular flux analysis, cells were refreshed with 525 µL experimental medium. Seventy-five microliters of 8-fold concentrated solution of *E. coli* O111:B4 LPS (C_final_ = 50 ng/mL), 9-fold concentrated solution of Oligomycin A (C_final_ = 1.47 µM), 10-fold concentrated solution of FCCP (C_final_ = 1.33 µM), and 11-fold concentrated solution of Antimycin A/Rotenone/2-Deoxyglucose (C_final_ = 1.21 µM/1.21 µM/20 mM) were injected sequentially at the indicated time points by the Seahorse XFe24 Analyzer. The injected compounds were prepared in the experimental medium.

The ECAR and OCR were normalized to cells numbers per treatment condition based on cell numbers from a parallel plate that were treated exactly the same way from day 1. The stabilized ECAR and OCR following the injection of Antimycin A/Rotenone/2-Deoxyglucose were used to derived glycolysis-dependent ECAR and oxidative phosphorylation (OxPhos)-dependent OCR. Glycolysis and OxPhos at the baseline and following LPS treatment, maximal glycolytic capacity, maximal respiratory chain capacity, maximal tricarboxylic acid (TCA) cycle capacity, and proton leak were calculated for individual traces as shown in **Supplementary Figure 4**.

### RNA Sequencing

dTHP-1 cells (1 × 10^6^ cells/well) were primed for 24 h with DCATH-2 in 6 well plates containing 1 ml medium, using culture medium as control. After priming, cells were washed 3 times and left to rest for 3 days in culture medium. Cells were stimulated for 6 h with 50 ng/ml *E. coli* O111:B4 LPS or fresh medium. Supernatants were stored for cytokine analysis. Cells were harvested by rinsing once with ice-cold DPBS and lysing cells in 100 µl RLT buffer (Qiagen, Hilden, Germany) containing 1% 2-mercaptoethanol. After scraping, per condition the contents of two wells were transferred into vials, flash frozen in liquid nitrogen and stored at -80°C for analysis. Total RNA was extracted in RLT buffer (RNeasy kit; Qiagen) supplemented with 2-mercaptoethanol and purified using a Qiagen QiaSymphony SP system. RNA libraries were prepared using the Truseq stranded total RNA (ribo-zero) library prep kit (Illumina, San diego, CA, USA) according to the manufacturer’s recommendations. RNA-sequencing was done with a NextSeq 500 system 1x 75 bp high-output kit (Illumina).

### RNA Sequencing Analysis

Single-end RNASeq reads were processed using the UMCU RNASeq pipeline (v2.3.0) with default settings. Read quality was assessed with FastQC (0.11.4) followed by splice-aware alignment against the human reference genome (GRCh37) with STAR (2.4.2a). RNA expression quantification was performed with htseq-count (0.6.0) in reverse-stranded mode. Differential gene expression analysis was carried out with the DESeq2 package in DEBrowser (https://debrowser.umassmed.edu/). In each comparison, genes were selected with an absolute fold-change > 2 and alpha P < 0.05. Pathway enrichment analysis was performed in g:Profiler (https://biit.cs.ut.ee/gprofiler/) using separate FDR ranked differentially expressed gene lists for up and down regulated genes with a Benjamini-Hochberg FDR correction for multiple testing and a FDR<0.05 threshold. Enriched pathways of GO Biologic Processes were visualized in Cytoscape (http://www.cytoscape.org/) using EnrichmentMap (http://www.baderlab.org/Software/EnrichmentMap) and a FDR cutoff of 0.01. Similarity statistic threshold was set at Jaccard>0.25 and filtered for gene set sizes between 5 and 500 genes (21).

### Statistical Analysis

Statistical analyses were performed using GraphPad Prism 8.4 (GraphPad Software, CA, USA). Differences between groups were calculated with one-way ANOVA using two-tailed Dunnett’s or Tukey’s multiple comparison tests. Alternatively, the Kruskal-Wallis with Dunn’s multiple comparisons test was used. Levels of significance were defined as p<0.05 (*), p<0.01 (**), p<0.001 (***) or p<0.0001 (****).

## Results

### Innate Immune Training of dTHP-1 Cells by CATH-2 Analogs

**Figure 1:**
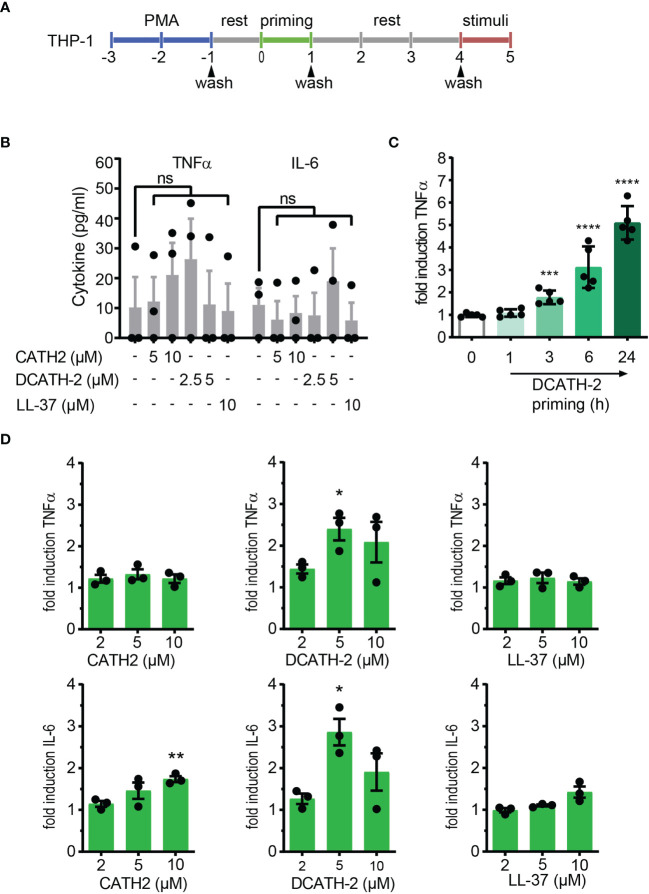
CATH-2 analogs induce a trained innate immune response to TLR2 and TLR4 agonists in dTHP-1 cells, leading to enhanced antimicrobial activity. **(A)** Schematic representation of *in vitro* THP-1 differentiation (100nM PMA) and training. **(B)** base levels of TNFα and IL-6 production by dTHP-1 cells after 24 h cathelicidin training, 3 days rest and no restimulation (means ± SEM; n=3). **(C)** Time-dependency of DCATH-2 training of dTHP-1 cells on TNFα production amplification in response to 24 h restimulation with 1 µg/ml Pam3CSK4. Representative experiment (means ± SD; n=5) of two experiments. Stimulated control cells: 372 ± 19 pg/ml TNFα. **(D)** Cathelicidin-trained dTHP-1 cells 24 h restimulated after 3 days rest with 10 ng/ml *S. minnesota* LPS (means ± SEM; n=3). Data were analyzed by one-way ANOVA with two-tailed Dunnett’s multiple comparison test against stimulated control cells. ns, not significant, *p < 0.05, **p < 0.01, ***p < 0.001, ****p < 0.0001.

Extrapolating from the trained immunity models in human monocytes (2, 3, 22), we examined if mature cathelicidin peptides could induce trained immunity in THP-1 cells, a human monocyte cell line. THP-1 cells were differentiated with 100 nM or 8 nM phorbol 12-myristate 13-acetate (PMA) during 48 h, washed 3 times and after overnight rest, primed with 2 -10 µM of chicken cathelicidin 2 (CATH-2), its full D- analog DCATH-2 or human cathelicidin LL-37 for 24 h. Subsequently, dTHP-1 cells were washed 3 times and restimulated after a 3-day rest period with TLR agonists (24 h) (**Figure 1A**). Basal production levels of TNFα and IL-6 were not altered by 24 h priming with 2.5-5 µM DCATH-2 or 5-10 µM CATH-2 (**Figure 1B**). DCATH-2 training of dTHP-1 cells was time-dependent, reaching the highest level of training after 24 h (**Figure 1C**) similar to training of primary monocytes with β-glucan (2). Training with 10 µM CATH-2 resulted in a 1.8-fold amplification of IL-6 production when restimulated with 10 ng/ml *Salmonella minnesota* LPS for 100 nM PMA dTHP-1 cells (**Figure 1D**). Priming with 5 µM of its full-D-amino acid analog DCATH-2 amplified the TNFα and IL-6 production 2- to 3-fold in response to LPS stimulation. Priming with 10 µM LL-37 did not significantly amplify IL-6 production in 100 nM PMA differentiated cells. Differentiation of THP-1 with a lower concentration of PMA has been suggested to result in lower basal levels of pro-inflammatory cytokine production (23) and might affect training efficiency. However, similar responses were found for CATH-2 and DCATH-2 training of 8 nM PMA differentiated THP-1 cells (**Supplementary Figure 1**).

### DCATH-2 Trained Immunity Amplifies Both TLR2 and TLR4 Activation

**Figure 2:**
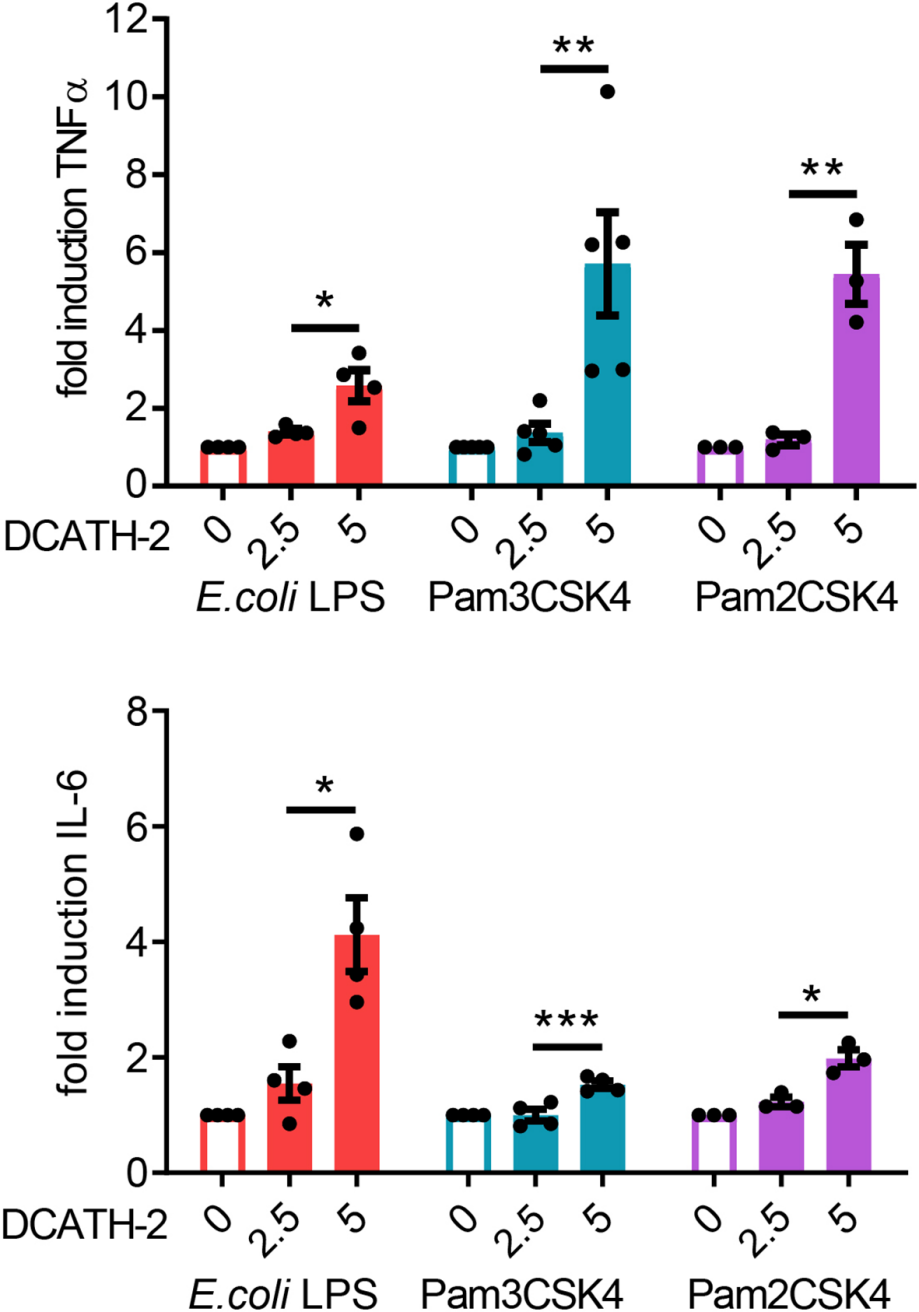
DCATH-2 training induced amplification of TNFα and IL-6 production in dTHP-1 cells in response to restimulation (24 h) with TLR2/4 agonists *E. coli* B4:O111 LPS (10 ng/ml; TLR4), Pam3CSK4 (1 µg/ml; TLR1/2) and Pam2CSK4 (1 µg/ml; TLR2/6). *E. coli* LPS stimulated control cells: 579 ± 119 pg/ml TNFα, 204 ± 49 pg/ml IL-6 (means ± SEM; n=4). Pam3CSK stimulated control cells: 647 ± 124 pg/ml TNFα, 662 ± 241 pg/ml IL-6 (means ± SEM; n=4). Pam2CSK stimulated control cells: 450 ± 124 pg/ml TNFα, 424 ± 197 pg/ml IL-6 (means ± SEM; n=3). Data were analyzed by one-way ANOVA with two-tailed Dunnett’s multiple comparison test against stimulated control cells. *p < 0.05, **p < 0.01, ***p < 0.001.

Next, we examined if DCATH-2 training affected the dTHP-1 response to different TLR2 and TLR4 ligands. dTHP-1 training with 5 µM DCATH-2 followed by restimulation with *E. coli* O111:B4 LPS, Pam3CSK4 or Pam2CSK4 resulted in amplified TNFα and IL-6 production (**Figure 2**). The production of CCL5 and CXCL10 was not amplified by DCATH-2 training (**Supplementary Figure 2**). Thus, stimulation by rough and smooth LPS (TLR4), triacyl- (TLR1/2) and diacyl lipopeptides (TLR2/6) all led to an amplified pro-inflammatory response by DCATH-2 trained dTHP-1 cells.

### DCATH-2 Trained dTHP-1 Cells Have Increased Antimicrobial Killing Capacity

**Figure 3:**
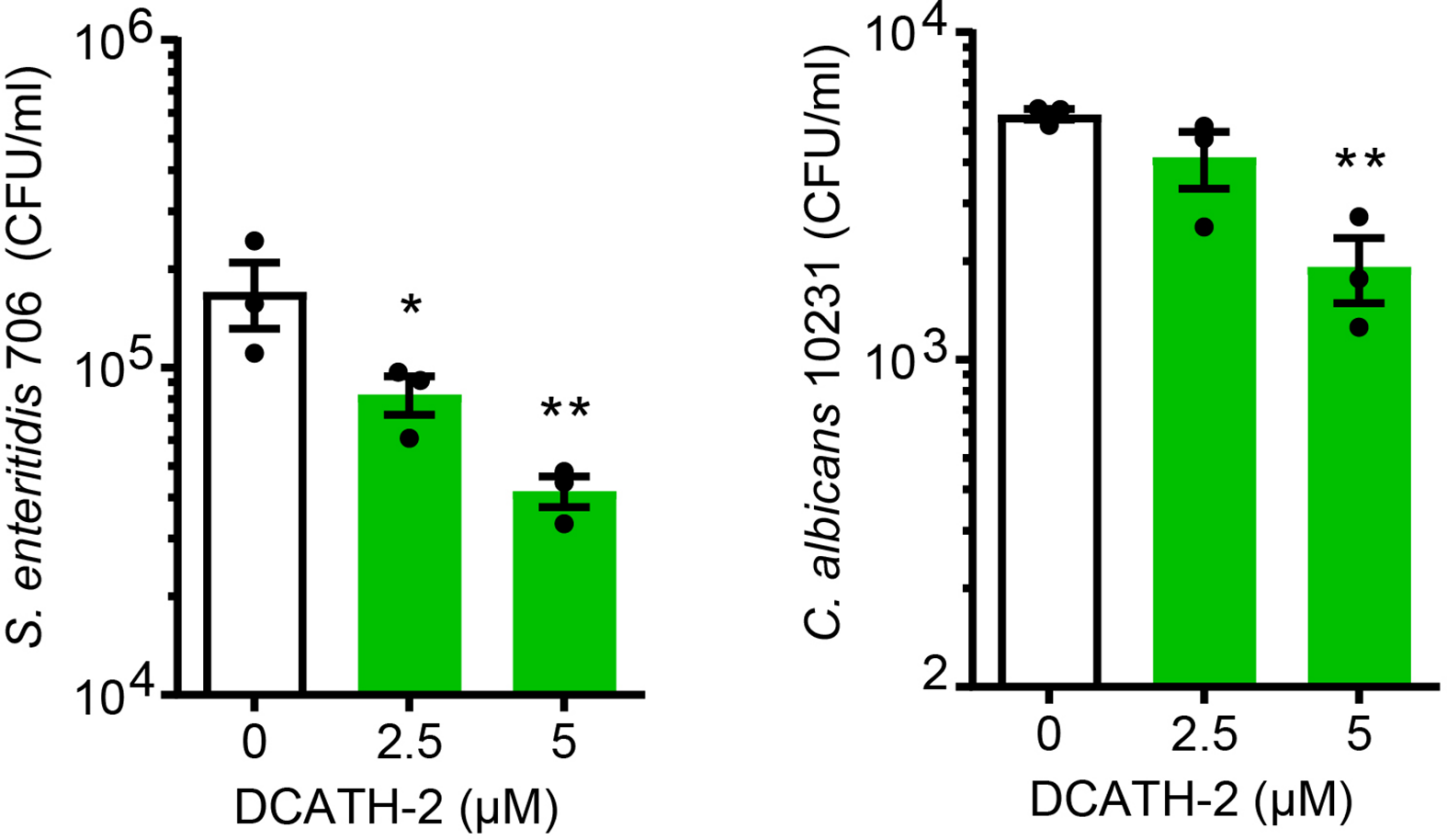
DCATH-2 trained dTHP-1 cells have increased antimicrobial killing capacity. Intracellular killing of *Salmonella enteritidis* 706 and candidacidal activity against *Candida albicans* ATCC10231 (means ± SEM; n=3). Data were analyzed by one-way ANOVA with two-tailed Dunnett’s multiple comparison test against stimulated control cells. *p < 0.05, **p < 0.01.

Intracellular pathogens attempt to evade or exploit the host innate immune system (24–26). We found that DCATH-2 training (24 h) of dTHP-1 cells enhanced their antimicrobial activity against *S. enteritidis* infection and *Candida albicans* infection (**Figure 3**). Compared to non-primed dTHP-1 cells, DCATH-2 primed dTHP-1 cells inhibited outgrowth of *S. enteritidis* after 24 h by 78 ± 11% and Candida albicans after 48 h by 68 ± 10%.

### DCATH-2 Training Utilizes the PI3K-mTOR-HIF1α Signaling Pathway

**Figure 4:**
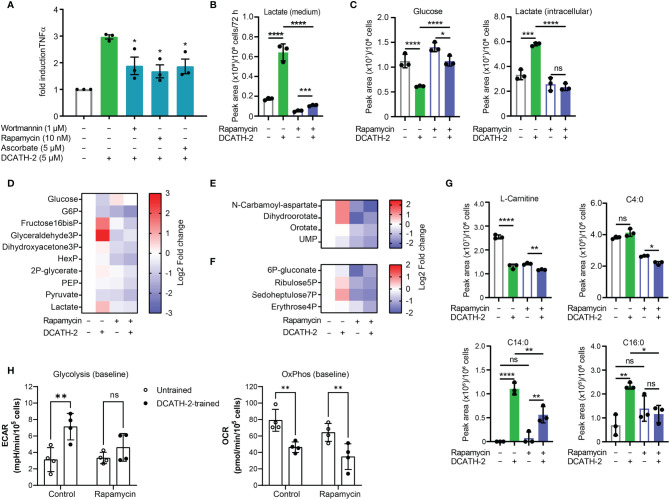
DCATH-2 training shifts dTHP-1 cell metabolism *via* mTOR towards aerobic glycolysis. **(A)** TNFα production of dTHP-1 cells primed with 5 µM DCATH-2 in the absence and presence of mTOR pathway inhibitors wortmannin, rapamycin and ascorbate after 3 days rest and 24 h restimulated with 1 µg/ml Pam3CSK4 (means ± SEM; n=4). Data were analyzed by one-way ANOVA with two-tailed Dunnett’s multiple comparisons tests. Pam3CSK stimulated TNFα production in control cells: medium, 304 ± 84 pg/ml; wortmannin, 354 ± 135 pg/ml; rapamycin, 318 ± 45 pg/ml; ascorbate, 366 ± 105 pg/ml (means ± SEM, n=3). **(B-G)** Representative metabolomics experiment (n=3) of two experiments in which dTHP-1 cells were trained with 5µM DCATH-2 without restimulation. dTHP-1 cell lysates and culture medium were obtained after DCATH-2 or medium priming followed by 3 days of rest. **(B)** Lactate production determined in dTHP-1 conditioned culture medium. **(C)** Intracellular glucose and lactate concentrations. **D-F** Heatmaps showing log2 fold changes in **(D)** glycolysis, **(E)** pyrimidine metabolism and **(F)** pentose phosphate pathways relative to unstimulated cells. **(G)** L-carnitine and acyl-carnitine levels (means ± SD). Data in panels **(B-G)** were analyzed by one-way ANOVA with two-tailed Tukey’s multiple comparisons tests. ns: not significant, *p < 0.05, **p < 0.01, ***p < 0.001, ****p < 0.0001. **(H)** Extracellular acidification rate (ECAR) and oxygen consumption rate (OCR) of dTHP-1 cells were measured 3 days after DCATH-2 and control training in a Seahorse XFe24 flux analyzer. Glycolysis-dependent ECAR and oxidative phosphorylation (OxPhos)-dependent OCR were calculated as indicated in **Supplementary Figures 4G, H**. n = 4 for each condition. Data in panel H were analyzed by two-way ANOVA with two-tailed Šidák’s multiple comparisons tests. *p < 0.05, **p < 0.01,***p < 0.001, ****p < 0.0001; ns, not significant.

Training of human monocytes is known to induce a shift in the cell metabolism from oxidative phosphorylation towards aerobic glycolysis mediated *via* the Akt-mTOR-HIF1α pathway in the case of β-glucan (6). To examine the involvement of the Akt-mTOR-HIF1α pathway in DCATH-2 training, dTHP-1 cells were pre-incubated with mTOR pathway specific inhibitors prior to priming with DCATH-2 peptide. Direct inhibition of PI3K (wortmannin), mTOR (rapamycin) and HIF1α (ascorbate) strongly interfered with DCATH-2 training of dTHP-1 cells (**Figure 4A**), indicating that DCATH-2 utilizes similar pathways as β-glucan to shift cell metabolism towards aerobic glycolysis. Indirect mTOR inhibition by AMPK activation with metformin and 5-aminoimidazole-4-carboxamide ribonucleotide (AICAR) during DCATH-2 training did not result in a significant reduction of Pam3CSK-induced TNFα production (**Supplementary Figure 3A**). Nor was DCATH-2 training inhibited by pan-Akt inhibitors triciribine or AZD5363 (**Supplementary Figure 3B**).

mTOR signaling controls the metabolism and activation of macrophages with mTORC1 upregulating rate-limiting enzymes in glycolysis, fatty acid synthesis and the pentose phosphate pathway (PPP) *via* SREBP1 and nucleotide synthesis *via* increased carbamoyl phosphate synthetase (CAD) activity, a rate-limiting enzyme in pyrimidine synthesis (27). To examine the impact of DCATH-2 training on dTHP-1 cells, metabolome analysis was performed using culture medium and cell lysates of DCATH-2 trained and untrained dTHP-1 cells after a 3-days rest and in the absence of TLR stimulation. To confirm mTOR involvement in the metabolomics experiment we tested in parallel the rapamycin inhibition of DCATH-2 training using TLR agonist induced TNFα and IL-6 production as a read-out. In this control experiment, the DCATH-2 amplified TNFα and IL-6 production upon *E. coli* LPS stimulation was strongly reduced by the presence of rapamycin during priming (**Supplementary Figure 3C**). As expected for increased aerobic glycolysis, DCATH-2 training augmented lactate secretion into medium (**Figure 4B**). In line with these results, intracellular lactate levels were higher and intracellular glucose concentrations were lower in DCATH-2 trained cells (**Figure 4C**). Analysis of metabolic intermediates indicated that DCATH-2 training increased glycolysis (**Figure 4D**), increased *de novo* pyrimidine synthesis (**Figure 4E**) and to a smaller extent the pentose phosphate pathway (**Figure 4F**), supporting involvement of enhanced mTOR activation. Rapamycin presence during DCATH-2 priming abolished augmentation of lactate production, glycolysis, pentose phosphate pathway and *de novo* pyrimidine synthesis (**Figures 4C-F**), confirming that DCATH-2 trained immunity is accompanied by an mTOR regulated shift towards aerobic glycolysis. The TCA cycle and urea cycle were not significantly affected by DCATH-2 training (**Supplementary Figures 3D, F** **)**. DCATH-2 training dramatically increased levels of intracellular medium-chain and long-chain acylcarnitines (**Figure 4G**) indicating decreased fatty acids utilization by the mitochondria. Next, we evaluated fluxes of glycolysis and oxidative phosphorylation by extracellular acidification rate (ECAR) and oxygen consumption rate (OCR). Consistent with the metabolomics findings, DCATH-2 training significantly increased glycolytic flux and suppressed the oxidative phosphorylation in mitochondria (**Figure 4H**). Furthermore, rapamycin treatment abolished the increase of glycolytic flux induced by DCATH-2 training.

### DCATH-2 Training-Inducing Metabolic Shift Is Maintained During LPS Stimulation

**Figure 5:**
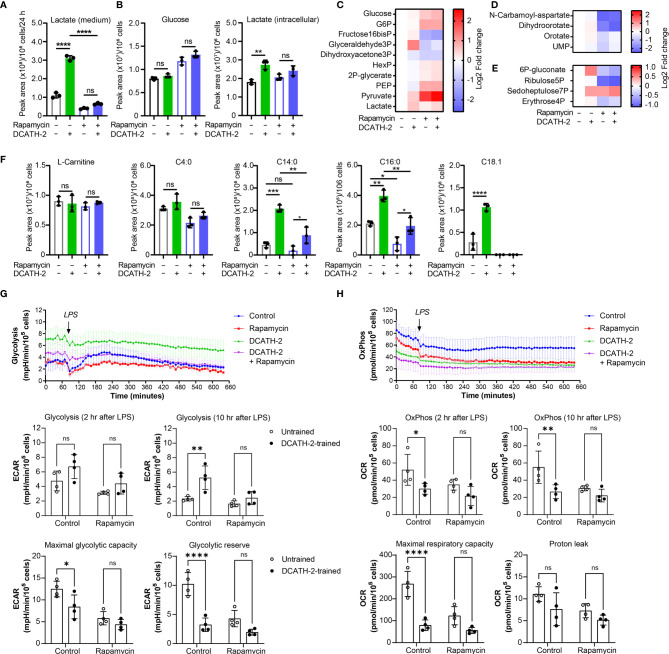
DCATH-2 training induced metabolic shift is maintained during LPS stimulation. Metabolomics experiment with 100 nM PMA differentiated dTHP-1 cells primed for 24 h with 5 µM DCATH-2 or medium in the presence or absence of 10 nM rapamycin followed by 3 times washing, 3 days of rest and 24 h stimulation with *E. coli* B4:O111 LPS (50 ng/ml; n=3). **(A)** Lactate production determined in dTHP-1 conditioned culture medium. **(B)** Intracellular glucose and lactate concentrations. **(C-E)** Heatmaps showing log2 fold changes in metabolites of **(C)** glycolysis, **(D)** pyrimidine biosynthesis and **(E)** pentose phosphate pathway relative to unstimulated cells. **(F)** L-carnitine and acyl-carnitine levels (means ± SD). Data were analyzed by one-way ANOVA with two-tailed Tukey’s multiple comparisons tests. ns: not significant, *p < 0.05, **p < 0.01, ****p < 0.0001. **(G)** Time course of glycolysis-dependent ECAR following the LPS treatment in dTHP-1 cells with or without rapamycin during DCATH-2 and control trainings. The bar charts summarize the glycolysis-dependent OCR after 2 h and 10 h LPS treatment, the maximal glycolytic capacity and the glycolytic reserve. **(H)** Time course of the OxPhos-dependent OCR following LPS treatment in dTHP-1 cells with or without rapamycin during DCATH-2 and control training. The bar charts summarize the OxPhos-dependent OCR after 2 h and 10 h LPS treatment, the maximal respiratory capacity and proton leak. The values are calculated as indicated in **Supplementary Figures 4G, H**. n = 4 for each condition. Data in **(G, H)** were analyzed by two-way ANOVA with two-tailed Šidák’s multiple comparisons tests. ns, not significant, *p < 0.05, **p < 0.01, ****p < 0.0001.

After 24 h LPS stimulation, DCATH2 trained cells still exhibited enhanced lactate production, increased glycolysis and PPP pathway metabolites, albeit at a lower extent (**Figures 5A-E**). Intracellular levels of medium-chain and long-chain acylcarnitines remained elevated in DCATH-2 trained cells (**Figure 5F**). Rapamycin reduced DCATH-2 training-induced effects on cell metabolism during LPS stimulation (**Figure 5**). LPS stimulation of DCATH-2 trained cells did not significantly affect the levels of metabolites in the TCA cycle, the urea cycle and amino acid metabolism (**Supplementary Figures 4A, C, E**). Thus, the DCATH-2 training induced metabolic shift is maintained during LPS stimulation. To confirm the metabolomics results, we monitored in real-time the changes in the fluxes of glycolysis and oxidative phosphorylation. In control cells, LPS induced a significant increase in glycolytic flux, which peaked at about 2 hours after stimulation and declined gradually in time (**Figure 5G**). In contrast, LPS did not induce a clear increase in glycolysis in DCATH-2-trained cells, which had an increased glycolytic flux already at baseline. Of note, while DCATH-2 trained cells are more glycolytic, their maximal glycolytic capacity was actually reduced and as a consequence they had less glycolytic reserve (**Figure 5G**). Rapamycin treatment effectively suppressed LPS-induced glycolytic flux in control cells. In addition, rapamycin also decreased glycolytic flux in LPS-stimulated, DCATH-2-trained cells to the level of control-trained cells, supporting that DCATH-2 training promotes a glycolytic phenotype *via* mTOR signaling.

Analysis of the flux of oxidative phosphorylation showed that, in the presence of LPS, DCATH-2-trained cells also had lower oxidative phosphorylation (**Figure 5H**) and the maximal respiratory capacity (**Figure 5H**) was also significantly lower than that of control cells, while the proton leak was not affected by DCATH-2 or rapamycin treatment (**Figure 5H**). Co-treatment with rapamycin abolished the differences in oxidative phosphorylation and maximal respiratory capacity between DCATH-2- and control-trained cells. Together, our data showed that the DCATH-2 training induced metabolic shift is maintained during LPS stimulation and involves mTOR signaling.

### DCATH-2 Training of dTHP-1 Cells Is Epigenetically Regulated

**Figure 6:**
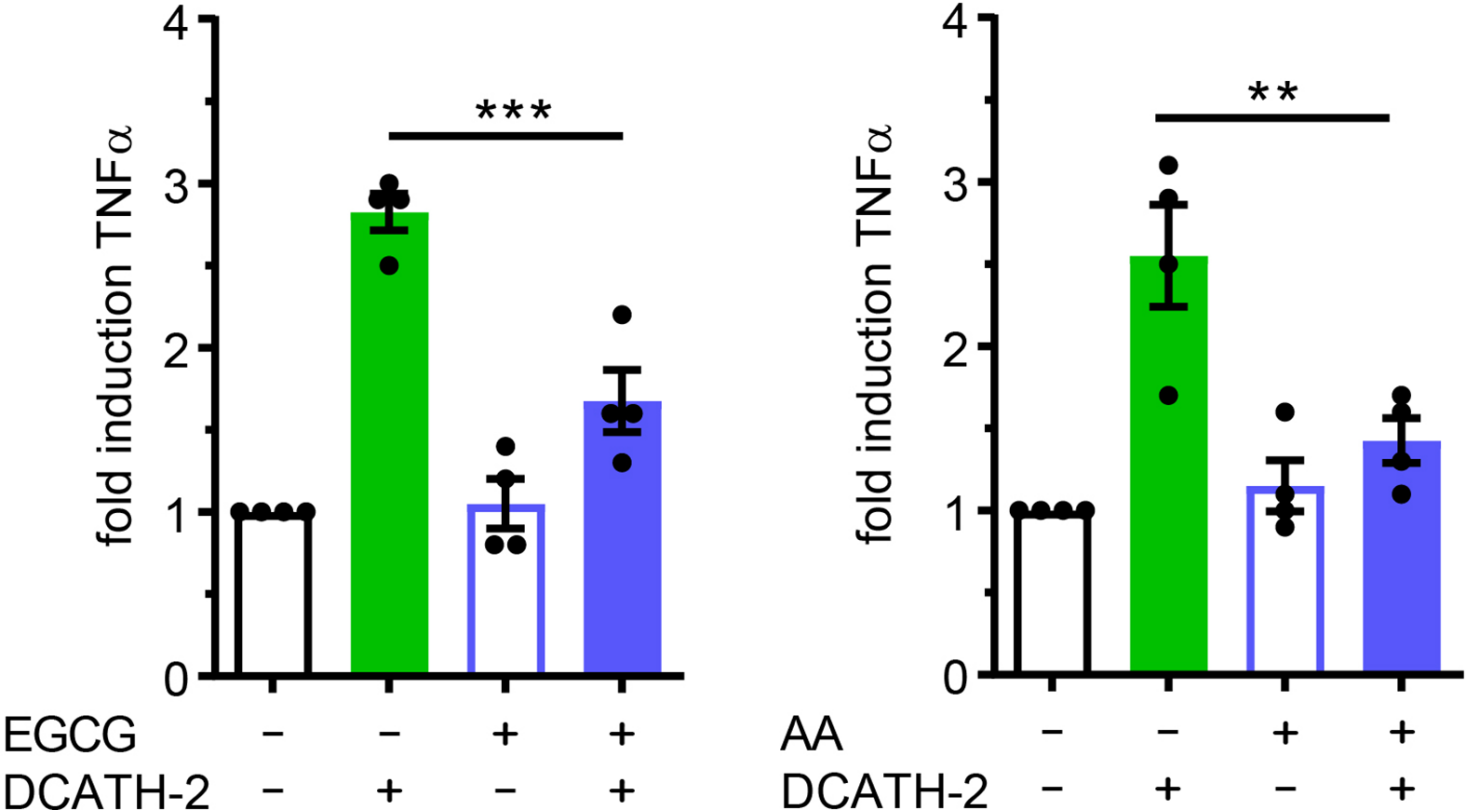
DCATH-2 training of dTHP-1 cells is epigenetically regulated. TNFα production of dTHP-1 cells primed with 5 µM DCATH-2 in the absence and presence of histone acetylation transferase inhibitors epigallocatechin gallate (EGCG) and anacardic acid (AA) after 3 days rest and restimulated (24 h) with 1 µg/ml Pam3CSK4 (means ± SEM; n=4). Stimulated control cells: 290 ± 41 pg/ml; EGCG and 287 ± 38 pg/ml; AA TNFα. Data were analyzed by one-way ANOVA with two-tailed Dunnett’s multiple comparison tests. **p < 0.01, ***p < 0.001.

DCATH-2 induced trained immunity augmented TLR2/4 ligand induced TNFα and IL-6 production without changing basal TNFα and IL-6 production, suggesting that DCATH-2 priming induced epigenetic reprogramming of dTHP-1 cells. Enriched H3K4me3 levels at promoters of immune-related genes including TNFα and IL-6 were found for human monocytes trained with BCG (3), β-glucan (2) or oxidized LDL (22) which positively related to enhanced transcription upon secondary stimulation. Increased H3K27ac levels in cytokine encoding genes were found in monocytes isolated after BCG vaccination and in β-glucan trained monocytes (6, 28). Neither methyltransferase inhibitor MTA (5’-methylthioadenosine), nor MLL1 inhibitor MM-102 prevented amplification of Pam3CSK4-induced TNFα production in DCATH-2 primed cells (**Supplementary Figures 5A, B** **)**. To determine the role of histone acetylation in DCATH-2 training, dTHP-1 cells were primed in the presence of histone acetyltransferase (HAT) inhibitors curcumin (10 µM), garcinol (10 µM), epigallocatechin gallate (EGCG, 40 µM) and anacardic acid (50 µM). It was found that DCATH-2 training of dTHP-1 cells was abrogated by histone acetyl transferase (HAT) inhibitors EGCG and anacardic acid (**Figure 6**) but not by garcinol or curcumin (**Supplementary Figures 5C, D** **)**. HAT inhibitors have different specificities, i.e., garcinol and curcumin inhibit p300 and PCAF (29, 30), whereas EGCG and anacardic acid inhibit Tip60 as well as p300 and PCAF (31, 32). Additionally, Tip60 expression is upregulated by garcinol (33). Taken together this suggest a role for MYST HAT members in DCATH-2 trained immunity. Histone acetylation requires cytosolic acetyl-CoA as substrate that is primarily produced by conversion of citrate to oxaloacetate by ATP citrate lyase. Although DCATH-2 training did not significantly change total acetyl-CoA levels or the citrate/oxaloacetate ratio (**Supplementary Figure 6** **)**, both were dramatically reduced by rapamycin, suggesting that rapamycin may interfere with epigenetic reprogramming by globally affecting acetyl-CoA metabolism.

### Trained Immunity Induced by DCATH-2 Requires MAPK p38 Signaling

**Figure 7:**
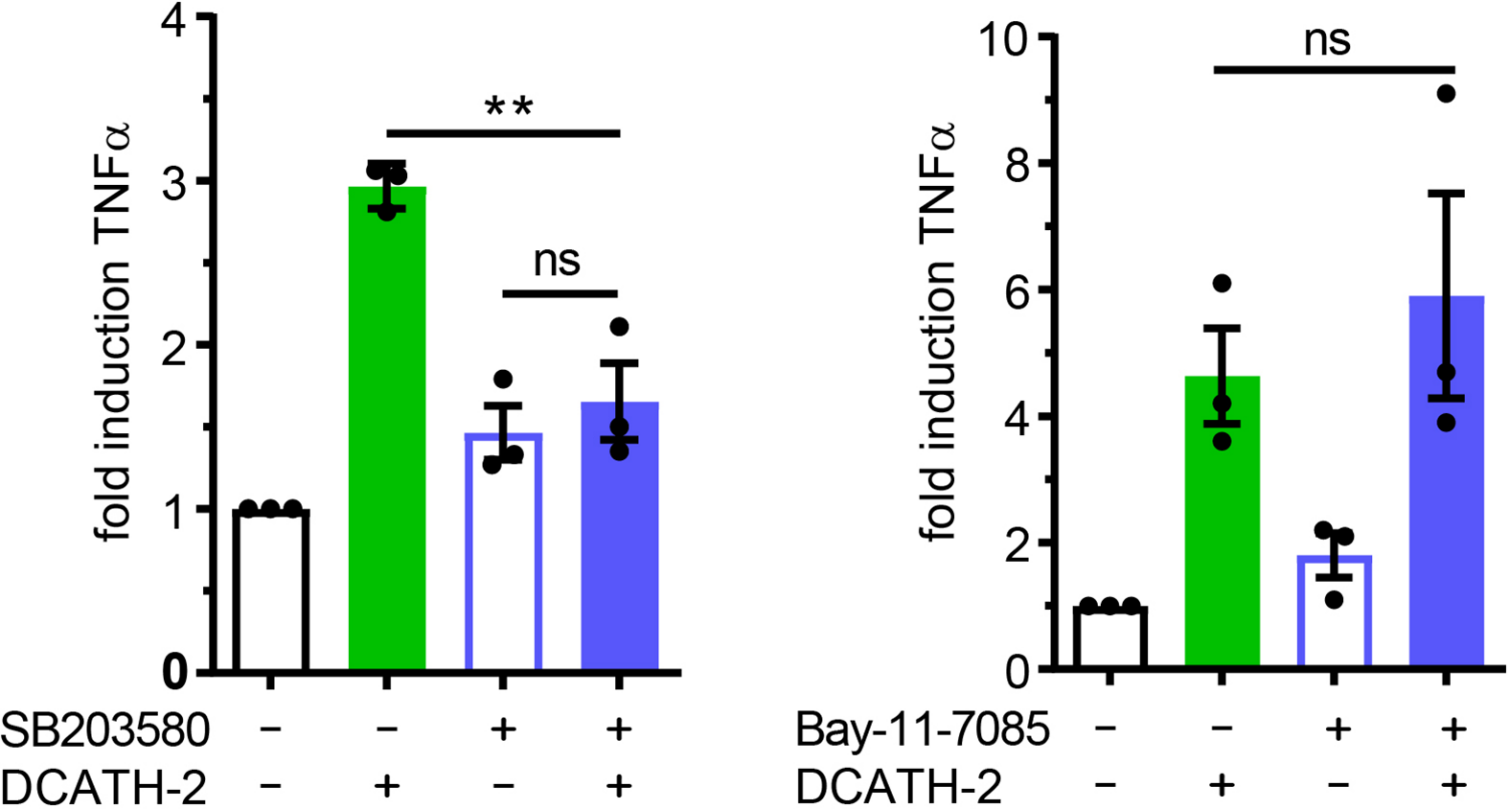
Trained immunity induced by DCATH-2 requires MAPK p38 signaling. TNFα production of dTHP-1 cells primed with 5 µM DCATH-2 in the presence and absence of inhibitors of MAPK p38 (SB203580) and NFκB (Bay-11-7085) after 3 days rest and restimulated (24 h) with 1 µg/ml Pam3CSK4 (means ± SEM; n=3). Stimulated control cells: 304 ± 84 pg/ml; p38 and 261 ± 24 pg/ml; NFκB TNFα. Data were analyzed by one-way ANOVA with two-tailed Dunnett’s multiple comparison tests. **p < 0.01. ns, not significant.

Both p38 and ERK are important signaling pathways for regulation of pro-inflammatory cytokines such as TNFα (34, 35) and IL-6 (36). MAPK p38-mediated signaling is involved in trained immunity with β-glucans (1, 2). To define the involvement of MAPKs in the DCATH-2 training, dTHP-1 cells were incubated with the specific inhibitors of p38 (SB203580), ERK (PD58059) and JNK (SP600125). Inhibition of MAPK p38 completely blocked the effect of DCATH-2 training that enhanced Pam3CSK-induced TNFα production (**Figure 7**), whereas ERK and JNK inhibition had no effect (**Supplementary Figure 7**). Pre-incubation with 10 µM NF-κB inhibitor Bay-11-7085 did not impair DCATH-2 training of dTHP-1 cells (**Figure 7**).

### D-CATH-2 Training of dTHP-1 Cells Is Mediated by Purinergic Receptors

**Figure 8:**
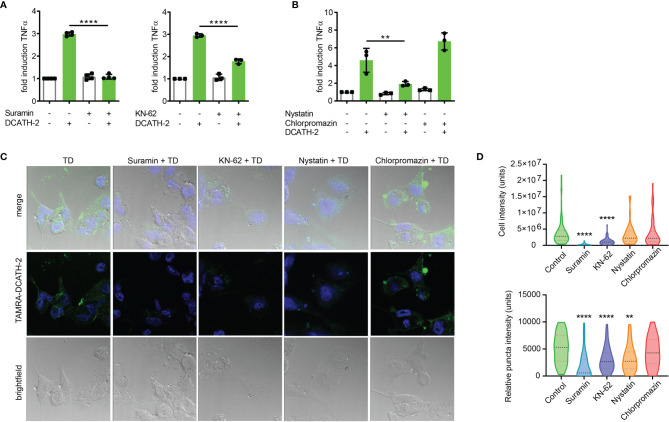
D-CATH-2 training of dTHP-1 cells is mediated by purinergic receptors. **(A)** TNFα production of dTHP-1 cells primed with 5 µM DCATH-2 in the absence and presence of broad spectrum P2R inhibitor suramin (means ± SEM; n=4) and P2X7R-specific inhibitor KN-62 (means ± SEM; n=3) after 3 days rest and restimulated (24 h) with 1 µg/ml Pam3CSK4. **(B)** TNFα production of dTHP-1 cells primed with 5 µM DCATH-2 in the absence and presence of lipid raft/caveolae-mediated endocytosis inhibitor nystatin and clathrin-mediated endocytosis inhibitor chlorpromazine (means ± SEM; n=3) after 3 days rest and restimulated (24 h) with 1 µg/ml Pam3CSK4. Stimulated control cells: 294 ± 46 pg/ml (suramin), 306 ± 53 pg/ml (KN-62) and 260 ± 24 pg/ml (endocytosis) TNFα. Data in panels **(A, B)** were analyzed by one-way ANOVA with two-tailed Dunnett’s multiple comparison tests. **(C)** Confocal imaging analysis of the uptake of TAMRA-labelled D-CATH-2 peptide by dTHP-1 cells in the presence of purinergic receptor and endocytosis inhibitors suramin, KN-62, nystatin and chlorpromazin. **(D)** Violin plots of internalized TAMRA-DCATH-2 peptide in the absence and presence of purinergic receptor and endocytosis inhibitors, expressed as total intensity per cell and puncta intensity per cell (median indicated by dotted line; n=3). Data were analyzed by Kruskal-Wallis with Dunn’s multiple comparisons test. **p < 0.01, ****p < 0.0001.

Immunomodulatory functions of cathelicidins have been associated with different receptors such as epidermal growth factor receptor (EGFR), formyl peptide receptor (FPR) and purinergic receptor P2X7R (20). Although P2X7R activation is primarily triggered by high levels of ATP, other endogenous ligands including the cathelicidin LL-37 are able to interact and activate P2X7R (37). To examine the involvement of P2X7R in cathelicidin training of monocytes, dTHP-1 cells were pre- and co-incubated with the P2 family inhibitor suramin or with P2X7 inhibitor KN-62. Suramin completely blocked the effect of DCATH-2 training on production of TNFα, whereas this was partially inhibited by P2X7R inhibitor KN-62 (**Figure 8A**). Internalization of LL-37 by monocytes is known to be P2X7-dependent and occurs *via* clathrin- and caveolae/lipid raft-mediated endocytosis (20). A function of the P2X7 receptor in training may therefore be to facilitate uptake of DCATH-2 peptide by monocytes/macrophages. To test this hypothesis, dTHP-1 cells were pretreated with the endocytosis inhibitors nystatin (caveolae/lipid raft-mediated) and chlorpromazin (clathrin-mediated). Pam3CSK4-induced TNFα production by DCATH-2 trained cells was strongly impaired by nystatin treatment but not affected by chlorpromazin (**Figure 8B**) suggesting that DCATH-2 training of dTHP-1 cells requires uptake *via* lipid raft-mediated endocytosis. Confocal imaging analysis of TAMRA-labelled DCATH-2 uptake by dTHP-1 cells in the presence of suramin, KN-62 or nystatin showed a reduction of overall signal intensity in the presence of P2/P2X7R inhibitors and a reduced relative intensity in puncta for P2/P2X7R inhibitors as well as for caveola/lipid raft-mediated endocytosis (**Figures 8C, D** **)**. TAMRA-DCATH-2 uptake was not reduced by inhibition of clathrin-mediated endocytosis. These findings confirmed that DCATH-2 training correlates with caveolae/lipid raft-mediated uptake by dTHP-1.

### DCATH-2 Training Skewed Transcription Is Sustained During Restimulation

**Figure 9:**
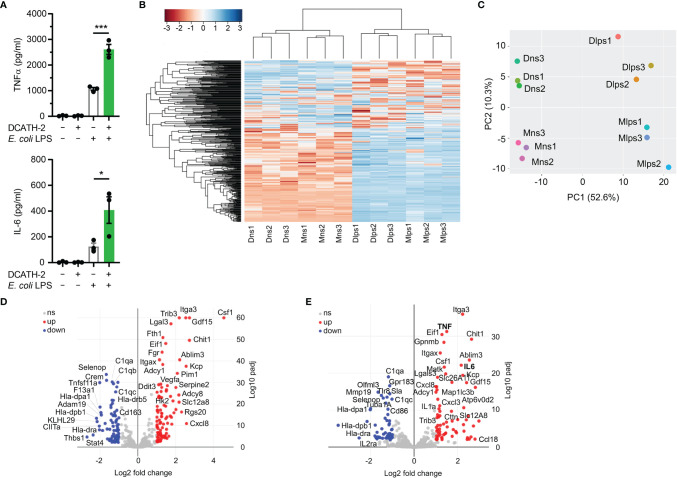
RNA sequencing of DCATH-2 trained dTHP-1 cells, primed for 24 h with 5 µM DCATH-2 or medium followed by 3 times washing, 3 days of rest and 6 h restimulation with *E. coli* B4:O111 LPS or medium. **(A)** TNFα and IL-6 production after restimulation. Paired two-tailed students t-tests against stimulated control cells. *p < 0.05, ***p < 0.0001. **(B)** Heatmap of differentially expressed genes (DEGs) with FDR<0.01 and minimal 2-fold increase relative to controls. Conditions: Mns, unstimulated control cells; Dns, unstimulated DCATH-2 trained cells; Mlps, LPS-stimulated control cells; Dlps, LPS-stimulated DCATH-2 trained cells. **(C)** Principal component analysis of all DEGs. **(D)** Volcano plot of DEGs in DCATH-2 trained dTHP-1 cells in absence of restimulation (Dns *vs* Mns) relative to control cells. **(E)** Volcano plot of DEGs in DCATH-2 trained THP-1 cells after LPS restimulation (Dlps *vs* Mlps) relative to control cells. ns, non-significant.

To elucidate which biological processes were persistently altered by DCATH-2 training of dTHP-1 cells, the transcriptome (RNA-seq) of DCATH-2 primed cells was compared with that of control cells after 3 days rest and after 6 h LPS stimulation. LPS stimulation resulted in a 2.5-fold and 3.3-fold amplification of TNFα and IL-6 production (**Figure 9A**). Hierarchical clustering of RNA-seq data showed subsets of genes that remained altered by DCATH-2 priming after 3 days rest (**Figure 9B**). Principal component analysis indicated that most variation between differentially expressed genes (DEGs) could be explained by LPS stimulation (52.6%) and DCATH-2 training (10.3%) (**Figure 9C**). 156 up- and 126 downregulated DEGs were identified for trained and rested dTHP-1 cells compared to unstimulated control cells. Following LPS stimulation, 71 upregulated and 54 downregulated DEGs were identified in DCATH-2 trained LPS-stimulated cells relative to LPS-stimulated control cells. Comparison of genes using volcano plots (**Figures 9D, E** **)** revealed a subset of genes uniquely upregulated/induced by LPS stimulation in trained cells (**Supplementary Table 1**) and were related to events occurring extracellularly and at the plasma membrane and associated with enhanced cellular responses to cytokine stimulus, cytokine-mediated signaling and cytokine/chemokine receptor binding. **Supplementary Figure 8** shows the DCATH-2 induced modest upregulation of mTORC2-regulated genes, amino acid transporter transcription and epigenetic enzymes after training and a 3-day rest.

### Pathway Analysis of Biological Processes Influenced by DCATH-2 Training

**Figure 10:**
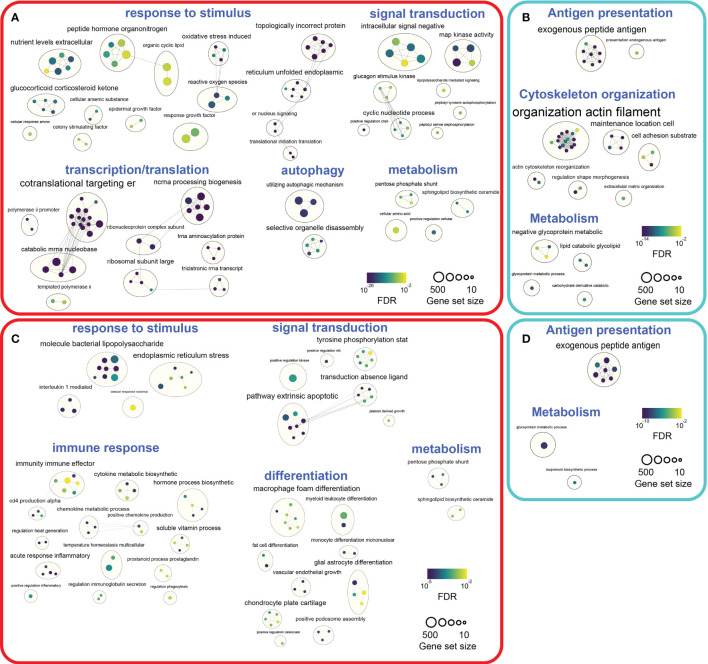
Pathway enrichment analyses of DCATH-2 trained unstimulated and LPS stimulated dTHP-1 cells. **(A)** Enrichment maps of GO biological processes upregulated in trained unstimulated dTHP-1 cells. **(B)** Enrichment maps of GO biological processes downregulated in trained unstimulated dTHP-1 cells. **(C)** Enrichment maps of GO biological processes upregulated in LPS-stimulated trained dTHP-1 cells. **(D)** Enrichment maps of GO biological processes downregulated in LPS-stimulated trained dTHP-1 cells. Ranked gene list of differentially expressed genes (FDR<0.01) were analyzed for enriched pathways using g:Profiler. Gene set sizes were filtered between 10 and 500 genes, nodes represent pathways with an FDR Q value cutoff <0.01 and connectivity (edges) were restricted by Jaccard cutoff of 0.25. AutoAnnotate was used to group clusters of similar pathways into major biological themes.

To map the biological processes altered by DCATH-2 priming after 3 days rest, pathway enrichment analysis was performed with G:profiler (38) and visualized using Cytoscape (39) and Enrichment Map (40). G:profiler analysis of differentially expressed genes revealed 590 upregulated and 612 downregulated GO biological processes (FDR<0.05) in DCATH-2 trained unstimulated dTHP-1 cells. Enriched pathways (FDR<0.01) were visualized separately using EnrichmentMaps in Cytoscape and clustered within themes using autoannotation. Major upregulated themes were cellular response to stimulus, transcription and translation. Minor upregulated themes were signal transduction, autophagy and metabolism (**Figure 10A** **;** **Supplementary Figure 9A** **;** **Supplementary Table 2**). Upregulated pathways were associated with oxidative stress, endoplasmic reticulum (ER) stress and unfolded protein response (UPR) linked to enhanced transcription and translation. Heatmaps of leading-edge gene expression confirmed upregulation of nonsense-mediated mRNA decay, PERK-mediated unfolded protein response (UPR), positive regulation of RNA polymerase II to stress and ribosome biogenesis. Most prominent downregulated processes were clustered around antigen processing and presentation (**Figure 10B** **;** **Supplementary Figure 9B** **;** **Supplementary Table 2**).

Next, we investigated how DCATH-2 training affected biological processes under inflammatory conditions. G:profiler analysis of DEGs (FDR<0.05) resulted in 322 upregulated and 416 downregulated GO biological pathways in DCATH-2 trained LPS-stimulated cells relative to untrained control cells. Enriched pathways (FDR<0.01) revealed upregulated biological processes associated with response to stimulus, signal transduction and immune response, differentiation and metabolism (**Figure 10C** **;** **Supplementary Figure 10A** **;** **Supplementary Table 3**). Downregulated pathways were related to antigen presentation and metabolism (**Figure 10D** **;** **Supplementary Figure 10B** **;** **Supplementary Table 3**).

## Discussion

We have previously shown that prophylactic treatment with DCATH-2 boosted immunity against bacterial infections (17, 18). However, the mechanisms that explain how a low dose of DCATH-2 can boost immunity despite a 10-day gap between administration *via* the amnion and infection 7 days post-hatching have so far remained unknown. A study by Hancock and co-workers on the cathelicidin-derived IDR-1 peptide indicated that monocytes and/or macrophages could be involved (41). Here, we demonstrate that some cathelicidins are indeed capable of inducing trained immunity in macrophages and map underlying biological processes involved in DCATH-2-induced trained immunity. Training with natural host defense peptide CATH-2 induced an enhanced production of IL-6 in dTHP-1 cells upon secondary stimulation with LPS. The full D-amino acid analog DCATH-2 elicited an even stronger effect and enhanced both TNFα and IL-6 production more strongly when restimulated with LPS or with TLR2 agonists.

Mechanistically, DCATH-2 training induces a metabolic switch that becomes apparent 4 days after priming, as has been reported for microbial ligands (6). Training reduces oxidative phosphorylation and increases aerobic glycolysis. In addition, metabolites of the pentose phosphate pathway and the *de novo* pyrimidine synthesis are increased. Consistent with the reduced flux through oxidative phosphorylation, and thus reduced oxidation of acetyl-CoA from fatty β-oxidation (FAO), DCATH-2 training leads to elevated levels of medium (C14:0) and long chain (C16:0) fatty acids. mTORC1 is a crucial metabolic regulator that integrates signals from nutrient availability, growth factors and cellular bioenergetic state. mTORC1 activation promotes glycolysis and lipid synthesis and inhibits lipolysis and FAO, resulting in intracellular lipid accumulation (42). Akt/mTOR/HIF1α signaling is also an integral part of β-glucan mediated trained immunity of macrophages (6). This suggests that mTORC1 could drive the observed metabolic switch in DCATH-2 trained macrophages. Indeed, we found that DCATH-2 training was partially dependent on mTOR, as the mTORC1 inhibitor rapamycin partially reversed the DCATH-2 induced increase in glucose uptake, glycolysis and lactate production.

Trained immunity is receptor mediated, i.e., β-glucan binds to dectin-1 (2), BCG activates intracellular receptor NOD2 (3), oxLDL interacts with OLR1 (28) and aldosterone signaling occurs *via* the mineralcorticoid receptor (43). Most endogenous alarmins interact with G-protein coupled receptors (GPCRs) at the cell surface thereby triggering multiple signaling pathways including PI3K/Akt, MAPK, PKC and small GTPases (8). Additionally, purinergic receptor P2X7 is known to be highly expressed on macrophages, is lipid raft associated (44) and regulates aerobic glycolysis *via* the Akt/PKB pathway (45). Although P2X7R is primarily activated by ATP, it can also be triggered by non-nucleotide agonists including polymyxin B and human cathelicidin LL-37 (46). In addition, P2X7R is involved in internalization of LL-37, which is associated with an enhanced clearance of intracellular bacteria (20). In our study, blocking of P2X7R or P2R activation resulted in respectively reduced and abrogated internalization of labeled DCATH-2 peptide that corresponded with a reduced and abrogated DCATH-2 trainings response, suggesting a key role for P2X7 in mediating trained immunity in macrophages. P2X7R-mediated endocytosis can be both clathrin-mediated and caveola/lipid raft-mediated (20). Here, inhibition of caveolae/lipid raft-mediated uptake but not clathrin-mediated uptake, inhibited DCATH-2 induced training and reduced relative puncta intensity of internalized labeled DCATH-2, but not overall cell intensity. This result is expected, as puncta represent internalized DCATH-2 trafficking to endosomes and lysosomes. In accordance with the antibacterial activity found for internalized LL-37 (20), DCATH-2 trained dTHP-1 cells exhibited augmented killing of *Salmonella enteritidis* and *Candida albicans*. Interestingly, P2X7R activation can be achieved by both L- and D-amino acid peptides, as was demonstrated for LL-37 and its full D-amino acid analog (37). This may be explained by conserved helicity within small peptides. Although D-peptides have inverted amide peptide bonds and left-handed helices instead of right-handed helices side chain topology is similar to the parental α-helical peptide (16). Helical interfaces feature in ~62% of protein complexes (47), while as much as ~80% of the FDA approved peptides consist of helical peptides (48). Although canonical receptor activation by D-amino acid-based peptides seems less likely, (D)-helical agonists of the GLP-1 (glucagon-like peptide) and PTH (parathyroid hormone) receptors were shown to equally well initiate adenylate-cyclase mediated PKA activation as their natural ligands, resulting in CREB activation (16). Thus, besides advantageous properties of high resistance against proteolytic degradation and low immunogenicity (16), D-amino acid peptides have a potential for non-canonical receptor activation. Considering that suramin pretreatment completely blocked DCATH-2 training, other P2 receptors than P2X7R are likely to be involved.

Surprisingly, we found that DCATH-2 training was PI3K-mediated, but Akt-independent. Akt-independent mTORC1 activation can occur by amino acid signaling *via* Rag GTPases and Rag-independent by glutamine (49). Additionally, prolonged rapamycin exposure, as is the case here, will inhibit mTORC2 as well (50), which activity at endosomes is PI3K-dependent (51) and, like mTORC1, can be activated by small GTPases and amino acids (52). mTORC2 activity promotes glycolysis and pentose phosphate pathways *via* c-Myc (53) and upregulates activities of amino acid transporters (53–55). This corroborates with the modest DCATH-2-induced transcription upregulation of mTORC2-regulated genes associated with glucose uptake (Slc2a1/Glut1, Slc2a3/Glut3), glycolysis (HK, PFK), the pentose phosphate pathway (G6PD, PGD) (52) and multiple amino acid transporters e.g., LAT1, xCT, Slc1a5, Slc6a9, Slc7a2, suggesting that also mTORC2 signaling may contribute to trained immunity in macrophages. Downstream, P2X7R activation in macrophages is linked to MAPK p38 and ERK signaling (56). In line with these findings, we found complete abrogation of DCATH-2 training when priming occurred in the presence of MAPK p38 inhibitor SB203580, but not by inhibitors of JNK or ERK signaling. As observed for β-glucan (2), MDP- and flagellin-mediated (1) trained immunity, MAPK p38 signaling plays a significant role in DCATH-2 trained immunity.

Trained immunity in human monocytes has been shown to alter chromatin epigenetic marks such as histone methylation (H3K4me1, H3K4me3) and acetylation (H3K27Ac) in enhancer and promoter regions of immune-related genes (2, 57). In line, the observed metabolic switch in macrophages provides metabolites necessary as substrates or cofactors for epigenetic modifications (58) and metabolite concentrations in part regulate chromatin modifier activities (59). In our model, pre-incubation with pan-methylation inhibitor MTA did not abolish DCATH-2 training of dTHP-1 cells. Similarly, long-term transcriptional memory in a B cell to macrophage differentiation model was found to be independent of H3K4 methylation (60). In this model, LPS restimulation was correlated to repressive histone H3K27me3 mark demethylation. Likewise, LPS-induced innate immune memory in macrophages involved a sustained reduction of repressive histone H3K9me2 marks (61). Modestly increased transcription of histone demethylases KDM6B and KDM3A (62) were also found in DCATH-2 trained cells, suggesting that a reduction of repressive epigenetic marks may contribute to DCATH-2 training.

To gain more insight into the biological processes adapted in DCATH-2 trained immunity, pathway enrichment analysis was done with RNA-sequence data obtained from DCATH-2-trained and control dTHP-1 cells. We found that transcription/translation was among the most prominently upregulated biological processes in unstimulated DCATH-2 trained cells. Modest upregulation was found for pathways associated with oxidative stress, endoplasmic reticulum (ER) stress, and the unfolded protein response (UPR). Overall, intracellular amino acid levels were lower in unstimulated trained cells compared to control cells, potentially reflecting increased usage of amino acid for protein synthesis which corresponded to increased ribosome biogenesis (GO:0042254). The increased need of trained cells for building blocks for RNA, DNA and protein synthesis and protein folding is expected to generate some level of ER stress as is seen for activation of macrophages (63). However, moderate dynamic changes in ER protein processing and folding can be dealt with by activating the UPR that determines cell fate by regulating signaling pathways linked to autophagy, apoptosis and inflammation (22). We found that DCATH-2 trained unstimulated cells were associated with modest mitophagy (GO:0000422). Similarly, autophagy related gene beclin-1 was upregulated in β-glucan trained mice but not in myeloid cell-specific HIF1a KO mice in which β-glucan trained immunity was abrogated (6). BCG-induced autophagy was also necessary for induction of trained immunity and protection against bladder cancer (64). Furthermore, enriched pathway analysis indicated positive regulation of sphingolipid biosynthesis in DCATH-2 trained cells, which in RAW264.7 cells was found to be directly linked to autophagy (65). Upregulation of sphingolipid biosynthesis occurred in unstimulated cells and LPS-stimulated DCATH-2 trained cells.

In conclusion, we show that the endogenous alarmin cathelicidin-2 induces trained immunity in macrophages and that this property can be reinforced by using a stable D-amino acid analog of cathelicidin-2. Our findings indicate that trained immunity is a plausible explanation for the protective effect of DCATH-2 peptide in the chicken and zebrafish embryonic infection models. As expected for an endogenous inducer of trained immunity the pro-inflammatory responses evoked by (D)CATH-2 training remain relatively subtle. Since HDPs are released by local epithelial cells and/or recruited neutrophils at the site of infection, part of their alarmin function could be to facilitate innate immune memory. Further research is necessary to investigate the potential of trained immunity induced by natural, locally released alarmins *in vivo*. Moreover, identification of biomarkers correlated to endogenous alarmin-induced trained immunity is pivotal in development of alarmin-based therapeutics as alternatives to antibiotics.

## Data Availability Statement

RNA-seq data have been deposited in the ArrayExpress database at EMBL-EBI (www.ebi.ac.uk/arrayexpress) under accession number E-MTAB-11601. The complete dataset can be accessed here https://www.ebi.ac.uk/arrayexpress/experiments/E-MTAB-11601. The Metabolomics data have been deposited to the EMBL-EBI MetaboLights database with the identifier MTBLS4495. The complete dataset can be accessed here https://www.ebi.ac.uk/metabolights/MTBLS4495.

## Author Contributions

AD and HH designed the study. AD, JA, AB, and NE performed the experiments. AH performed mass spectrometry analysis. J-CC and AD performed the Seahorse experiments. AH, J-CC, and CB performed analysis of metabolomics data. MN provided expertise for innate immune training experiments. AD, EV, MS, and HH analyzed the data. AD wrote the paper. All authors contributed to the article and approved the submitted version.

## Funding

This work was supported by the Dutch Ministry of Economic Affairs *via* the Immuno Valley Alternatives to Antibiotics (ALTANT) program (Animal-Specific Immunomodulatory Antimicrobials 2 project), NWO-TTW grant 14924 to the Bac-Vactory program and a TKI-LSH allowance grant (project no. LSHM18040). MN was supported by an ERC Advanced Grant (#833247) and a Spinoza grant of the Netherlands Organization for Scientific Research.

## Conflict of Interest

AD, EV, and HH are coinventors on two patent applications describing CATH-2 analogs as antimicrobial and immunomodulatory anti-infectives for veterinary therapy which are currently licensed for product development by Zoetis and one patent application pending describing innate immune memory stimulated by CATH-2 derivatives. In addition, AD, EV, HH, and MS have one CATH-2 based patent application pending describing CATH-2 analogs inhibition of *Streptococcus suis*.

The remaining authors declare that the research was conducted in the absence of any commercial or financial relationships that could be construed as a potential conflict of interest.

## Publisher’s Note

All claims expressed in this article are solely those of the authors and do not necessarily represent those of their affiliated organizations, or those of the publisher, the editors and the reviewers. Any product that may be evaluated in this article, or claim that may be made by its manufacturer, is not guaranteed or endorsed by the publisher.
