# Supplementary material for: Innate Immune Training of Human Macrophages by Cathelicidin Analogs
Source: Front Immunol. 2022 Jul 26;13:777530. doi: 10.3389/fimmu.2022.777530 (PMC9360325; doi:10.3389/fimmu.2022.777530)
Supplement: Supplementary file 4 [file DataSheet_4.pdf]

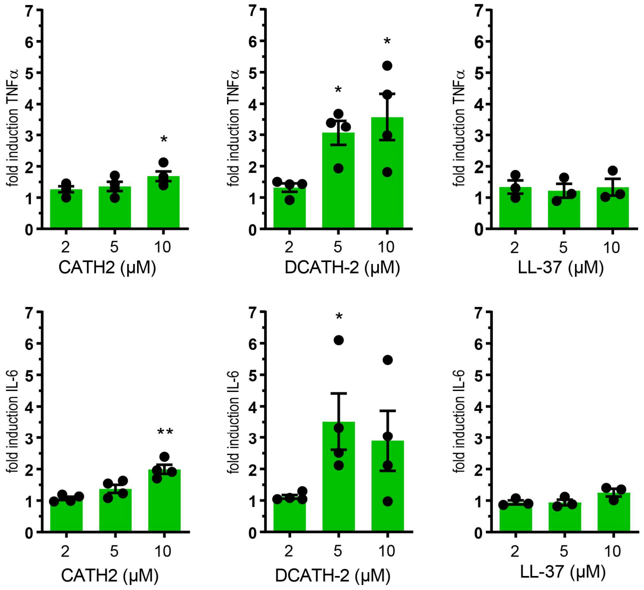

FIGURE S1 | Cathelicidin-induced trained immunity in 8nM PMA differentiated THP-1 cells. TNFα and IL-6 production of THP-1 cells differentiated with 8nM PMA and trained with different cathelicidin peptides after washing, 3 days rest and re-stimulation with 10 ng/ml *S. enterica* LPS. Means ± SEM from at least 3 independent experiments. LPS-stimulated control cells: 285 ± 45 pg/ml TNFα, 172 ± 22 pg/ml IL-6. * $p<0.05$, ** $p<0.01$. Data were analyzed by one-way ANOVA with two-tailed Dunnett's multiple comparisons tests against stimulated control cells.

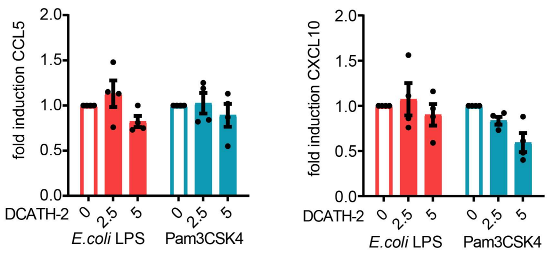

FIGURE S2 | DCATH-2 training did not induce amplification of CCL5 or CXCL10 production in dTHP-1 cells in response to restimulation (24 h) with TLR2/4 agonists *E. coli* B4:O111 LPS (10 ng/ml; TLR4) and Pam3CSK4 (1 µg/ml; TLR1/2). *E. coli* LPS stimulated control cells: 6.3 ± 2.0 ng/ml CCL5, 16.8 ± 3.3 ng/ml CXCL10 (means ± SEM; n=4). Pam3CSK stimulated control cells: 6.0 ± 3.5 ng/ml CCL5, 3.8 ± 0.6 ng/ml CXCL10 (means ± SEM; n=4). Data were analyzed by one-way ANOVA with two-tailed Dunnett's multiple comparison test against stimulated control cells. * $p<0.05$, ** $p<0.01$, **** $p<0.0001$.

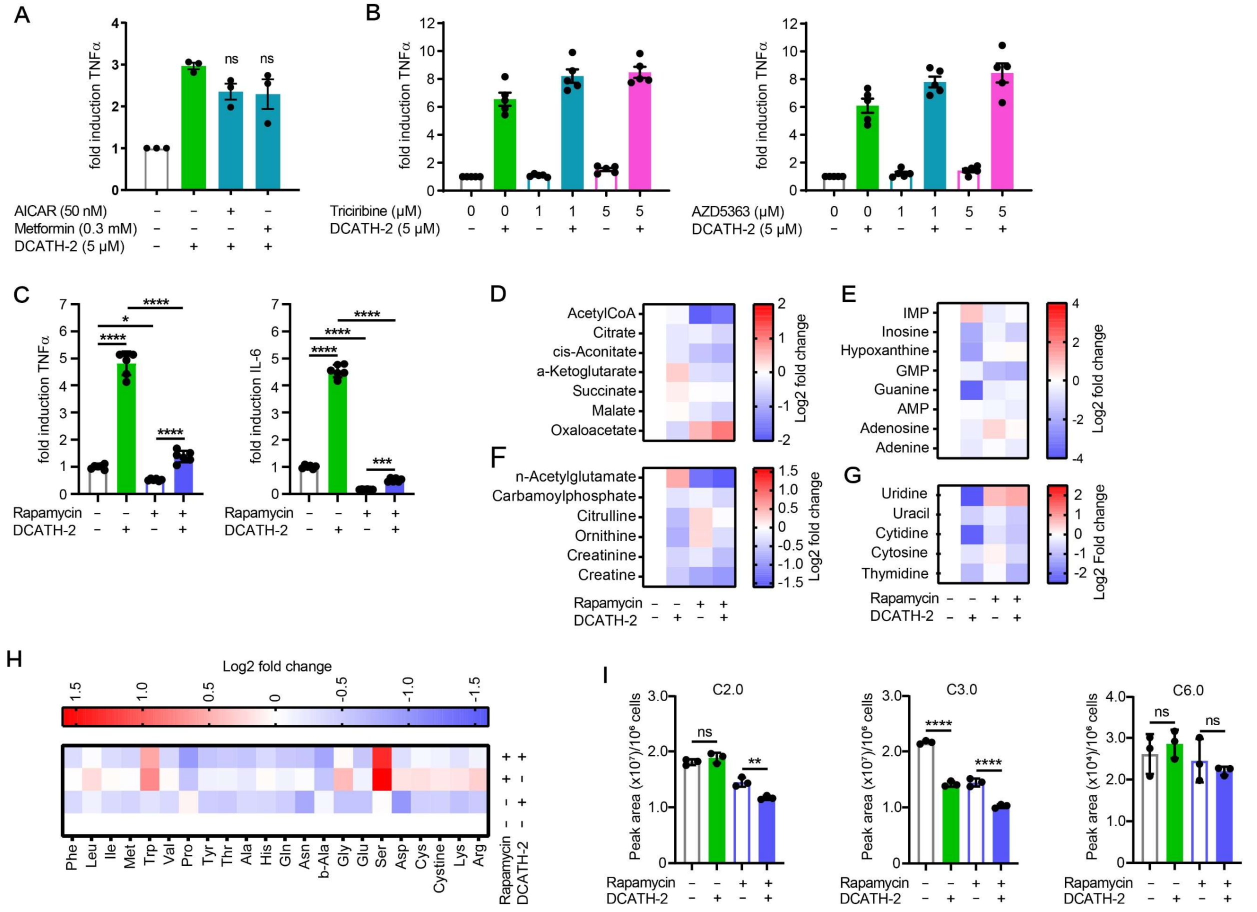

FIGURE S3 | DCATH-2 training shifts dTHP-1 cell metabolism towards aerobic glycolysis. (**A**) AMPK activation by AICAR of metformin does not significantly inhibit the amplified TNFα production in DCATH-2 trained dTHP-1 cells when restimulated with 1 µgPam3CSK4 after 3 days rest (means ± SEM; n=3). Data were analyzed by one-way ANOVA with two-tailed Dunnett's multiple comparisons tests. ns: not significant. Pam3CSK stimulated TNFα production in control cells: medium, 304 ± 84 pg/ml; AICAR, 335 ± 90 pg/ml; metformin, 387 ± 99 pg/ml. (**B**) preincubation with AKT inhibitors triciribine and AZD5363 do not prevent amplified TNFα production after DCATH-2 trained dTHP-1 cells (means ± SEM; n=6). (**C**) TNFα and IL-6 production of dTHP-1 cells trained with DCATH-2 in the in the absence and presence of mTOR inhibitor rapamycin (10 nM) after 3 days of rest when re-stimulation with *E. coli* B4:O111 LPS (50 ng/ml, means ± SD; n=6). (**D-I**) Representative metabolomics experiment (n=3) of two experiments with 100 nM PMA dTHP-1 cells. Cell lysates and culture medium were obtained after DCATH-2 or medium priming (24 h) followed by 3 days of rest. (**D-H**) Heatmaps show log2 fold changes in metabolites of the (**D**) TCA cycle, (**E**) purine synthesis, (**F**) urea cycle, (**G**) pyrimidine metabolism and (**H**) amino acid metabolism relative to medium primed unstimulated control cells. (**I**) Acylcarnitine levels. Means ± SD. ** p<0.01, **** p<0.0001. Data were analyzed by one-way ANOVA with two-tailed Tukey's multiple comparisons test.

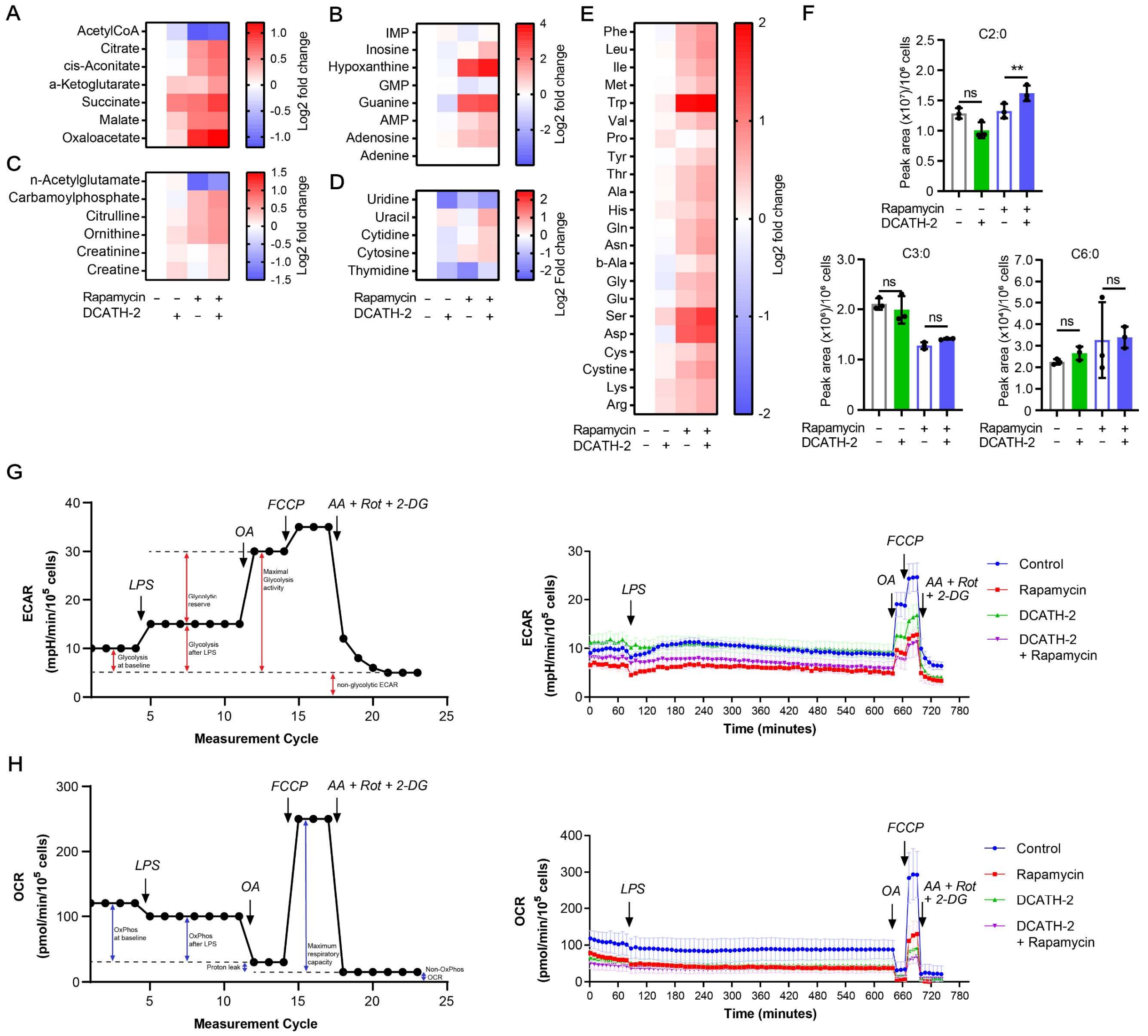

FIGURE S4 | DCATH-2 training induced metabolic shift is maintained during LPS stimulation. Metabolomics experiment (n=3) with 100 nM PMA differentiated dTHP-1 cells. Cell lysates and culture medium were obtained after DCATH-2 or medium priming (24 h) followed by 3 days of rest and 24 h stimulation with *E. coli* B4:O111 LPS. (**A-D**) Heatmaps showing log2 fold changes in metabolites of the (**A**) TCA cycle, (**B**) purine synthesis, (**C**) urea cycle, (**D**) pyrimidine metabolism and (**E**) amino acid metabolism relative to medium-primed LPS-stimulated cells. (**F**) Acylcarnitine levels. Means ± SD. ** p<0.01. Data were analyzed by one-way ANOVA with two-tailed Tukey's multiple comparisons test. Extracellular flux analysis determined in a XF24 Seahorse analyzer with explanation (left) and complete traces (right) of (**G**) the extracellular acidification rate (ECAR) and (**H**) the oxygen consumption rate (OCR). LPS, oligomycin A (OA), Carbonyl cyanide-p-trifluoromethoxyphenylhydrazone (FCCP), and a mixture of antimycin A (AA), rotenone (Rot), and 2-deoxyglucose (2-DG) were injected sequentially as indicated by the arrows. The data were normalized to cell numbers.

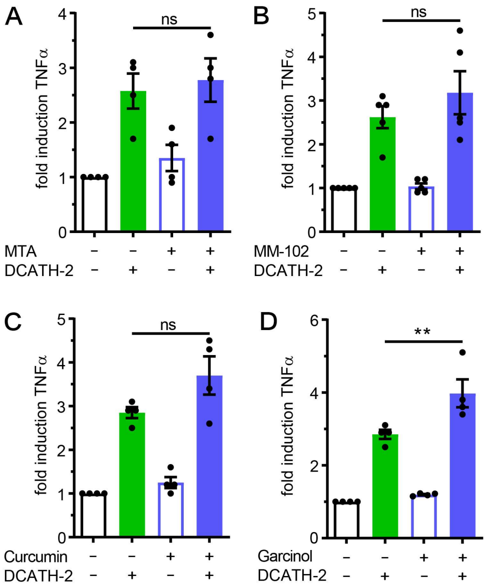

FIGURE S5 | DCATH-2 training is not affected by histone methyltransferase inhibitors. dTHP-1 cells were primed with 5 µM DCATH-2 in the absence or presence of (**A**) broad-spectrum histone methyltransferase (HTM) inhibitor 5'-methylthioadenosine (MTA) and (**B**) MML1 specific HTM inhibitor, MM-102, histone acetyltransferase inhibitors (**C**) curcumin and (**D**) garcinol, before 3 days resting and re-stimulation with 1 µg/ml Pam3CSK4. Pam3CSK-induced TNFα production in the absence of inhibitors: (**A**) 328 ± 45 pg/ml (means ± SEM, n=4); (**B**) 290 ± 37 pg/ml (means ± SEM, n=5); (**C, D**) 251 ± 4 pg/ml (means ± SEM, n=4). ns: not significant. ** $p<0.01$. Data were analyzed by one-way ANOVA using two-tailed Dunnett's multiple comparisons test.

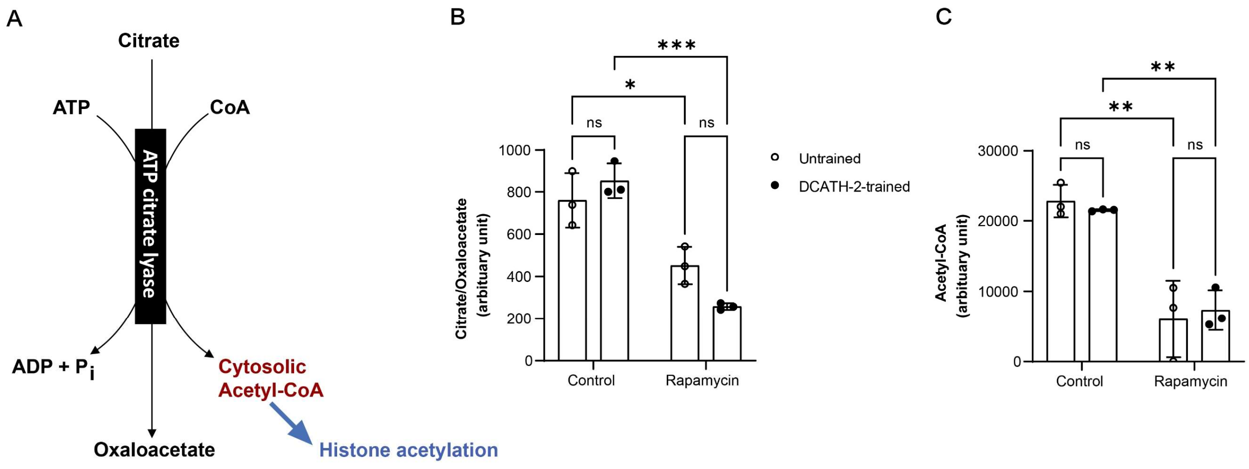

FIGURE S6 | Rapamycin reduces cytosolic acetyl-CoA production. (**A**) Diagram of cytosolic acetyl-CoA production needed for histone acetylation. The reduction of the citrate/oxaloacetate ratio (**B**) and acetyl-CoA production (**C**) by rapamycin. Data were derived from metabolomics experiment 2 (Supplementary file S1) and analyzed by 2-way ANOVA with two-tailed Šidák's multiple comparisons tests. * $p< 0.05$, ** $p<0.01$, ***, $p<0.001$.

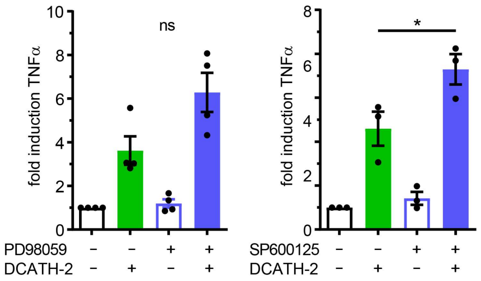

FIGURE S7 | DCATH-2 training of dTHP-1 cells is MAPK ERK and JNK-independent. Fold increase in TNFα production of dTHP-1 cells primed with 5 µM DCATH-2 in the absence or presence of MAPK ERK (PD98059) and JNK (SP600125) inhibitors, after 3 days rest and 24 h re-stimulation with 1 µg/ml Pam3CSK4. Pam3CSK4-stimulated control cells: 321 ± 58 pg/ml TNFα (ERK), means ± SEM, n=4; 260 ± 24 pg/ml TNFα (JNK), means ± SEM, n=3. ns: non-significant, * $p<0.05$. Data were analyzed by one-way ANOVA with two-tailed Dunnett's multiple comparisons test.

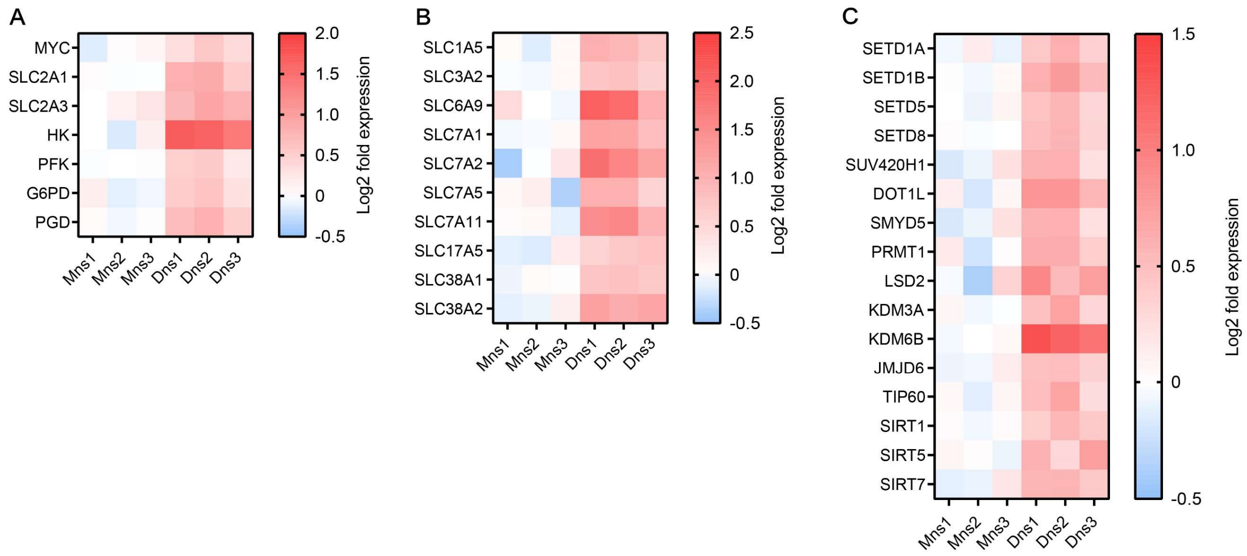

FIGURE S8 | Impact of DCATH-2 training on transcription of mTORC2-regulated genes, amino acid transporters and epigenetic enzymes. (**A**) Log2fold increase in transcription of mTORC2 regulated genes after DCATH-2 training and 3 days rest. (**B**) Log2fold increase in transcription of amino acid transporter genes after DCATH-2 training and 3 days rest. (**C**) Log2fold increase in transcription of epigenetic enzymes. Data were analyzed by two-tailed-t-test ($p < 0.05$).

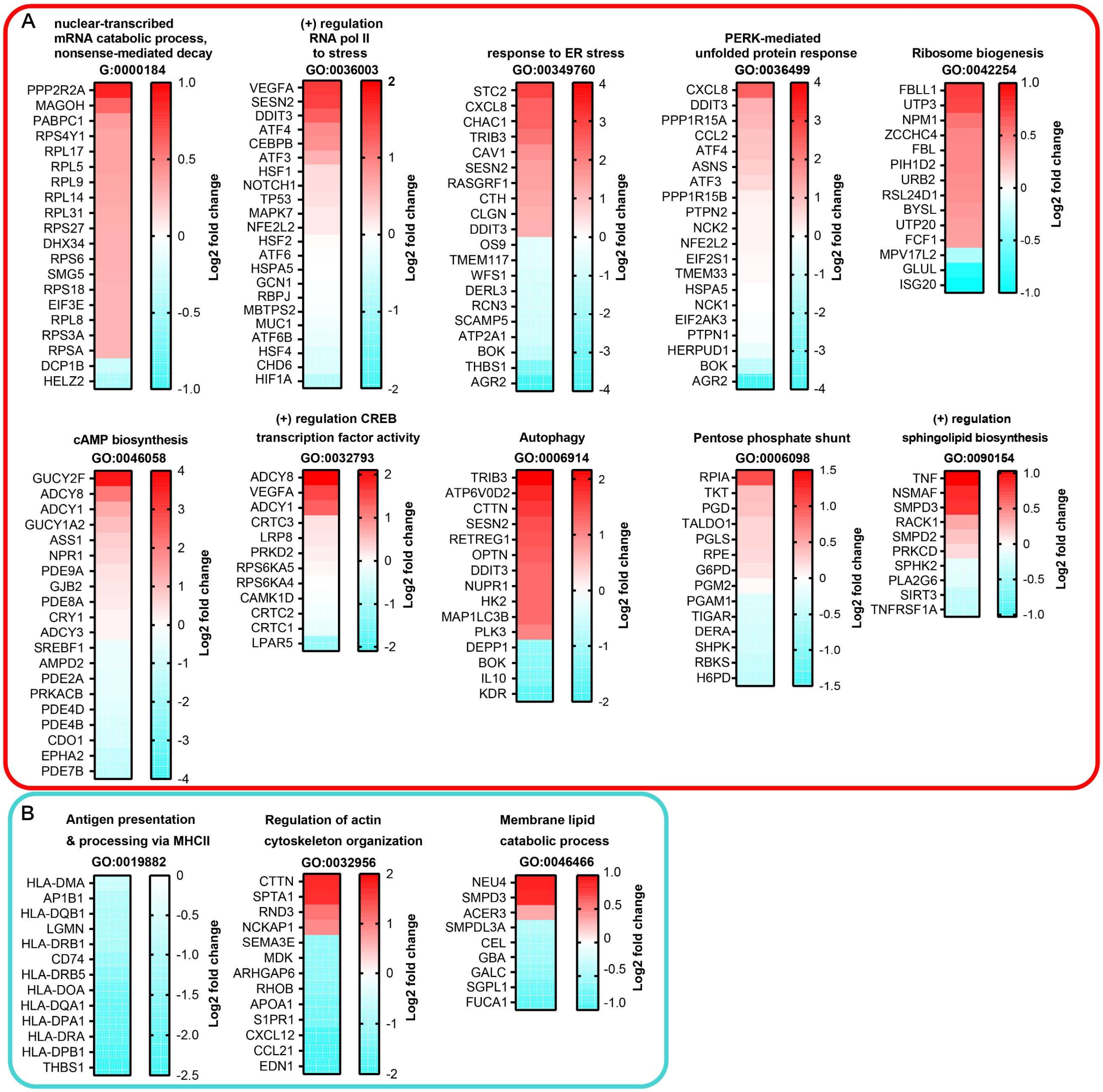

FIGURE S9 | Enriched biological processes in DCATH-2 primed unstimulated dTHP-1 cells. Expression values of leading-edge genes presented as log2 fold changes (FDR<0.01), mean values of 3 replicates. (**A**) Upregulated DEGs (**B**) downregulated DEGs.

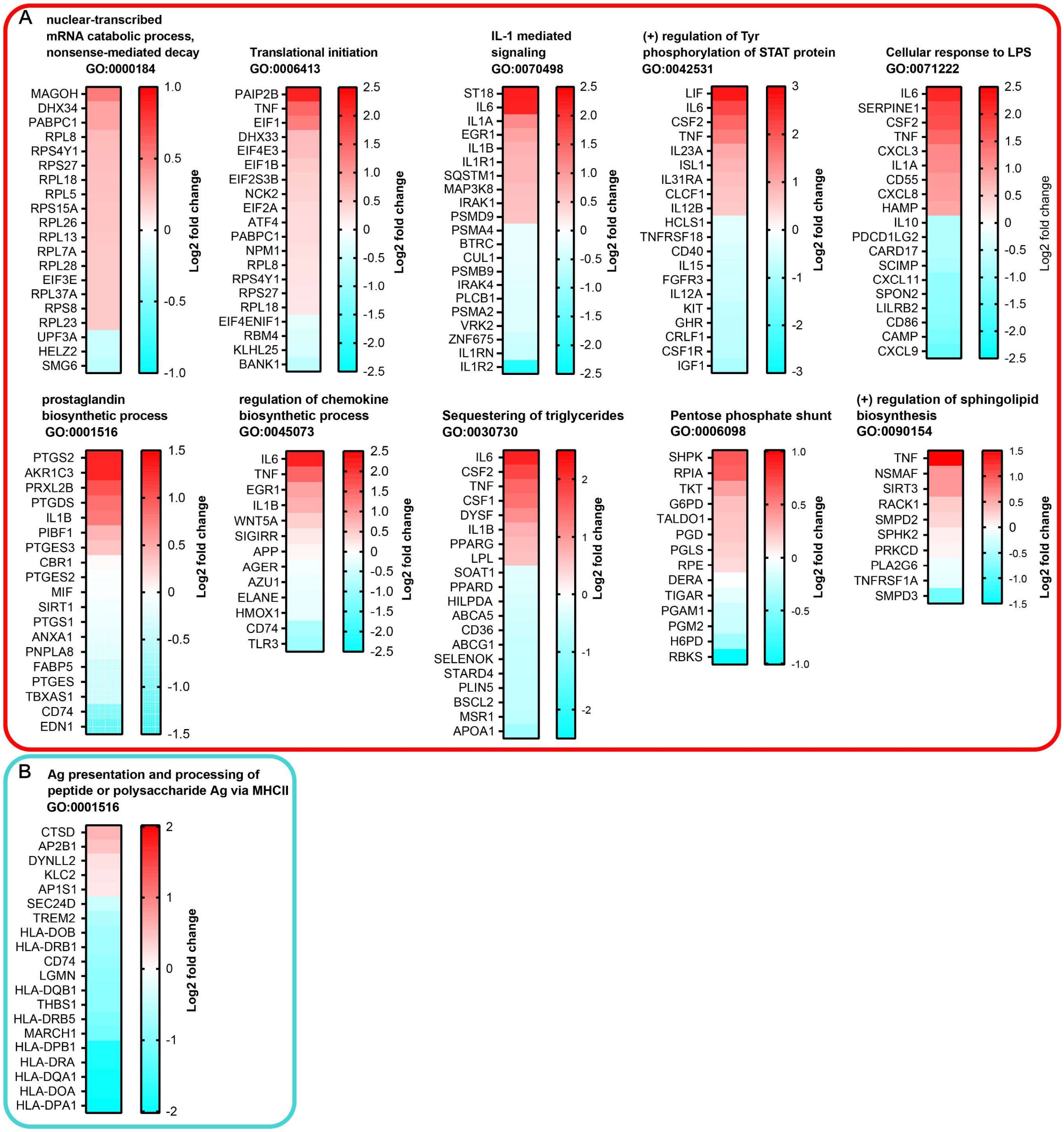

FIGURE S10 | Enriched biological processes in DCATH-2 primed LPS-stimulated dTHP-1 cells. Expression values of leading-edge genes presented as log2 fold changes (FDR<0.01), mean values of 3 replicates. (**A**) Upregulated DEGs (**B**) downregulated DEGs.

TABLE S1 | Genes uniquely upregulated after DCATH-2 training during LPS stimulation.

Gene	Description	Adjusted p-value	Log2 fold change
IL6	Interleukin-6	7.47E-23	2.17
MFSD2A	Major Facilitator Superfamily Domain Containing 2A	4.83E-17	1.04
CXCL3	C-X-C Motif Chemokine Ligand 3	6.93E-12	1.18
IL1A	Interleukin-1A	4.58E-11	1.15
TNFSF15	TNF Superfamily Member 15	2.24E-10	1.19
TRPM8	Transient Receptor Potential Cation Channel Subfamily M Member 8	5.84E-07	1.36
FOXF1	Forkhead Box F1	1.31E-06	1.21
LIF	LIF Interleukin 6 Family Cytokine	2.03E-06	2.73
MYLK	Myosin Light Chain Kinase	1.35E-05	1.17
CSF2	Colony Stimulating Factor 2	1.88E-05	1.81
CD22	CD22 Molecule	7.46E-05	1.18
TIE1	Tyrosine Kinase With Immunoglobulin Like And EGF Like Domains 1	1.61E-04	1.46
AHNAK2	AHNAK Nucleoprotein 2	1.77E-04	1.12
CCRL2	C-C Motif Chemokine Receptor Like 2	1.01E-03	1.04
ZNF358	Zinc Finger Protein 358	4.52E-03	1.04
HSPBAP1	HSPB1 Associated Protein 1	5.56E-03	1.06
SEPT3	Septin 3	6.92E-03	1.06
KLF2	Kruppel Like Factor 2	6.96E-03	2.54
CCL18	C-C Motif Chemokine Ligand 18	7.05E-03	2.81
FAM213B	Peroxiredoxin Like 2B	7.63E-03	1.02
TRIM47	Tripartite Motif Containing 47	8.53E-03	2.45
PTGS2	Prostaglandin-Endoperoxide Synthase 2	9.60E-03	1.29

TABLE S2 | Pathway enrichment analysis of DCATH-2 primed unstimulated dTHP-1 cells.

Cluster	Biological theme	Gene ontology	GO biological process	Gene set size	FDR Q-value
Upregulated					
cotranslational targeting ER	Translation	GO:0000184	Nu clear-transcribed mRNA catabolic process, nonsense-mediated decay	119	1.40E-26
polymerase ii promotor	Transcription	GO:0036003	positive regulation of transcription from RNA polymerase II promoter in response to stress	22	2.00E-04
ncrna processing biogenesis	Translation	GO:0042254	ribosome biogenesis	279	1.62E-09
topologically incorrect protein	Response to stimulus	GO:0034976	response to endoplasmic reticulum stress	253	3.04E-07
er nucleus signaling	Response to stimulus	GO:0036499	PERK-mediated unfolded protein response	20	5.55E-06
positive regulation creb	Signal transduction	GO:0032793	positive regulation of CREB transcription factor activity	12	2.27E-04
cyclic nucleotide process	Signal transduction	GO:0046058	cAMP metabolic process	14	4.80E-03
utilizing autophagic mechanism	Autophagy	GO:0006914	autophagy	452	1.90E-03
selective organelle disassembly	Autophagy	GO:0000422	autophagy of mitochondrion	70	4.94E-03
sphingolipid biosynthetic ceramide	Metabolism	GO:0090154	positive regulation of sphingolipid biosynthetic process	10	3.94E-03
pentose phosphate shunt	Metabolism	GO:0006098	pentose phosphate shunt	14	6.44E-03
Downregulated					
exogenous peptide antigen	Antigen presentation	GO:0019882	antigen processing and presentation	207	1.03E-08
organization actin filament	Cytoskeleton organization	GO:0032956	regulation of actin cytoskeleton organization	262	6.57E-06
Lipid catabolic glycolipid	Metabolism	GO:0046466	membrane lipid catabolic process	28	3.74E-03

TABLE S3 | Pathway enrichment analysis of DCATH-2 primed LPS-stimulated dTHP-1 cells.

**Cluster**	**Biological theme**	**Gene ontology**	**GO biological process**	**Gene set size**	**FDR Q-value**
Upregulated					
protein targeting er	Translation	GO:0000184	nuclear-transcribed mRNA catabolic process, nonsense-mediated decay	119	4.04E-05
protein targeting er	Translation	GO:0006413	translational initiation	184	4.04E-05
interleukin 1 mediated	Signal transduction	GO:0070498	interleukin-1 mediated signaling pathway	95	8.56E-04
tyrosine phosphorylation stat	Signal transduction	GO:0042531	(+) regulation of Tyr phosphorylation of STAT protein	44	4.28E-03
molecule bacterial lipopolysaccharide	Immune response	GO:0071222	cellular response to lipopolysaccharide	165	8.14E-05
Chemokine cytokine biosynthetic	Immune response	GO:0045073	regulation of chemokine biosynthetic process	13	1.29E-03
prostanoid process prostaglandin	Immune response	GO:0001516	prostaglandin biosynthetic process	19	8.84E-03
macrophage foam differentiation	Differentiation	GO:0030730	sequestering of triglyceride	11	9.52E-04
pentose phosphate shunt	Metabolism	GO:0006098	pentose-phosphate shunt	14	3.10E-03
sphingolipid biosynthetic ceramide	Metabolism	GO:0090154	positive regulation of sphingolipid biosynthetic process	10	7.44E-03
Downregulated					
exogenous peptide antigen	Antigen presentation	GO:0002495	antigen processing and presentation of peptide or polysaccharide antigen via MHC class II	89	1.41E-04
